# Progesterone level in assisted reproductive technology: a systematic review and meta-analysis

**DOI:** 10.1038/s41598-024-81539-z

**Published:** 2024-12-28

**Authors:** Yee Cherng Lim, Mukhri Hamdan, Abha Maheshwari, Ying Cheong

**Affiliations:** 1 Complete Fertility, Princess Anne Hospital, Level F, Coxford Road, Southampton, SO16 5YA UK; 2 Department of Obstetrics and Gynaecology, Universiti Malaya, 50603 Kuala Lumpur, Malaysia; 3 Aberdeen Centre of Reproductive Medicine, Aberdeen, UK; 4 Human Development and Health Unit, Institute of Life Sciences, Faculty of Medicine, University of Southampton, Southampton, SO16 6YD UK

**Keywords:** Assisted reproductive technology, Embryo transfer, Intracytoplasmic sperm injection, In vitro fertilization, Pregnancy outcomes, Serum progesterone, Endocrinology, Health care

## Abstract

Elevated progesterone (EP) or inadequate progesterone levels during ART cycle monitoring may lead to cycle cancellations or further progesterone supplementation, but practice varies. It remains controversial whether modifying clinical practice in the presence or absence of EP improves clinical outcomes. This systematic review aims to investigate if progesterone levels at different phases of fresh and frozen ART cycles influence pregnancy outcomes, in particular, that pertaining to day 3 versus day 5 embryo transfers. A systematic search of EMBASE, MEDLINE, CINAHL, PubMed, SCOPUS and Web of Science identified studies from the year 2000. We included studies with women undergoing fresh and frozen IVF/ICSI cycles; with extractable per woman data on pregnancy outcomes where serum progesterone measurement was performed. We excluded interventional studies that influence clinical decisions or studies with donor cycles. The Newcastle Ottawa Scale (NOS) was used to determine the risk of bias. The primary outcome was LBR, and the secondary outcomes were OPR, CPR and MR. PICOS study protocol was used to include non-randomized studies of interventions (NRSI). Analysis was done using RevMan5 and the studies were pooled using the DerSimonian and Laird for random effects meta-analysis. The study was registered with PROSPERO (registration ID CRD42022382423). 64 studies (N = 57,988 women) were included. In fresh cycles, there is no evidence that at baseline EP impacts LBR (P > 1.5 ng/ml, OR 0.76 [95% CI 0.39–1.49], 2 studies, N = 309) and CPR (P > 1.5 ng/ml, OR 0.81 [0.38–1.71], 2 studies, N = 309). EP at ovulation trigger is associated with a lower LBR (P > 1.0 ng/ml, OR 0.40 [0.23–0.69], 2 studies, N = 2805) and CPR (P > 1.0 ng/ml, OR 0.49 [0.42–0.58], 3 studies, N = 3323; P > 1.1 ng/ml, OR 0.66 [0.53–0.83], 2 studies, N = 2444; P > 1.2 ng/ml, OR 0.61 [0.39–0.96], 6 studies, N = 844; P > 1.5 ng/ml, OR 0.37 [0.17–0.81], 6 studies, N = 13,870; P > 2.0 ng/ml, OR 0.43 [0.31–0.59], 3 studies, N = 1949) with D3 embryo but not D5 [LBR (P > 1.5 ng/ml, OR 1.02 [0.74–1.39], 3 studies, N = 5174) and CPR (P > 1.5 ng/ml, OR 0.88 [0.67–1.14], 6 studies, N = 5705)]. We could not meaningfully meta-analyse studies on the day of egg collection in fresh cycles, embryo transfer in fresh cycles, at ovulation trigger or before ovulation in natural FET cycles and FET cycles due to significant study heterogeneity. We acknowledged the limitations on including studies post year 2000 and the exclusion of studies with multiple observations, which may result in inherent publication bias and some confounding factors uncontrolled for. In conclusion, in controlled ovarian stimulation, EP at baseline did not impact on LBR; EP at ovulation trigger is associated with a lower LBR for D3 but not for D5 embryo transfer. In FET cycles, as the studies were heterogeneous, we were unable to combine the data in a meaningful way. This review is sponsored by Complete Fertility and the Ministry of Health, Malaysia.

**Supplementary Information:**

The online version contains supplementary material available at 10.1038/s41598-024-81539-z.

## Introduction

Progesterone level can be elevated (EP) (follicular phase or at ovulation trigger) or inadequate (luteal phase), both of which may be linked to reduced pregnancy rates. The optimization of progesterone level is therefore a key focus in clinical practice.

**Fig. 1:**
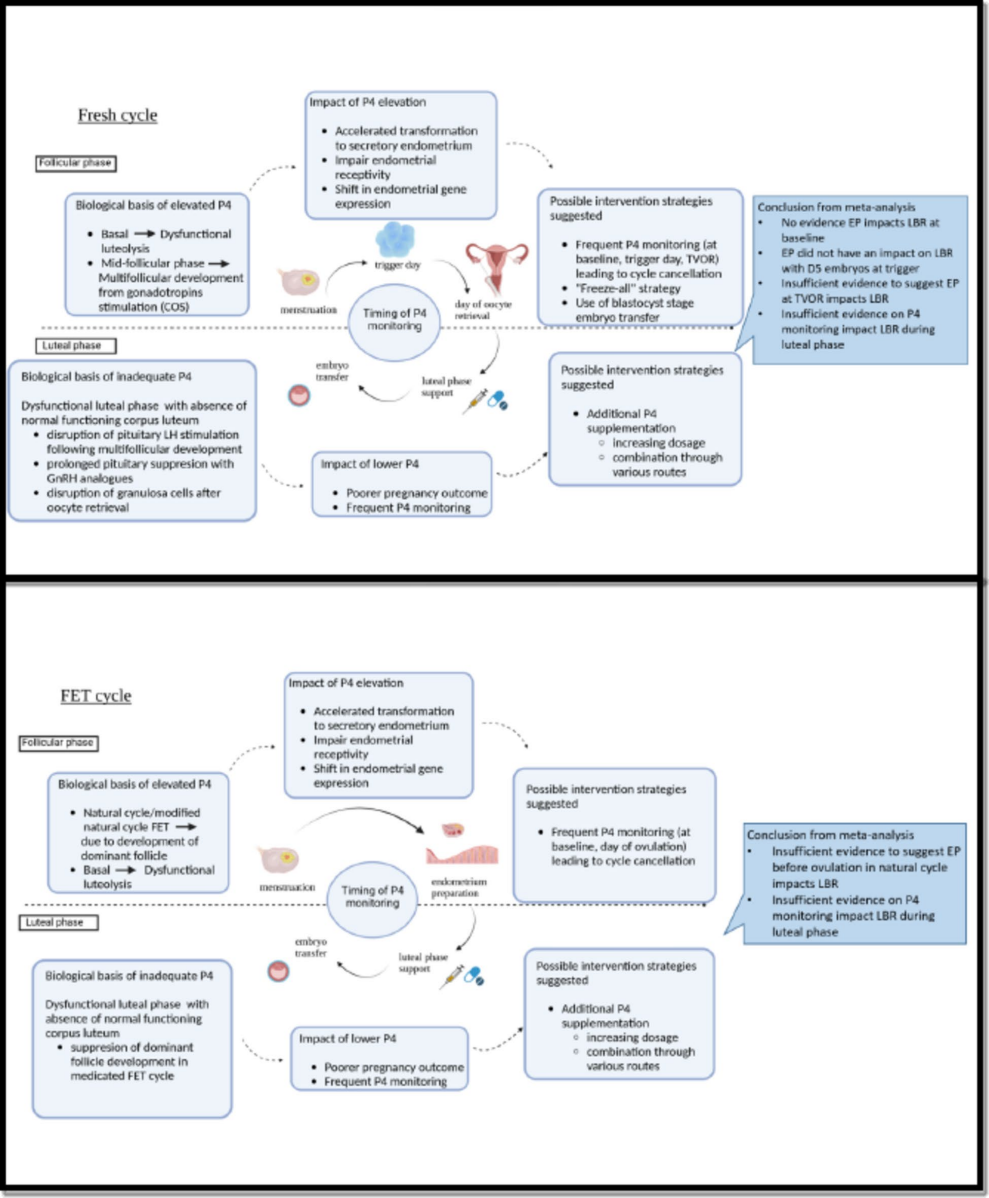
Biological basis and possible impact of progesterone monitoring in a fresh ovarian stimulation cycle and frozen embryo transfer cycle. *COS* controlled ovarian stimulation, *D5* day 5, *EP* elevated progesterone, *FET* frozen embryo transfer, *GnRH* gonadotropin-releasing hormone, *LBR* live birth rate, *LH* luteinising hormone, *P4* progesterone, *TVOR* transvaginal oocyte retrieval.

During ovarian stimulation, EP during the follicular phase up to the point of ovulation trigger, is postulated to cause premature advancement of the endometrium, thereby causing uterine embryo asynchrony and affecting endometrial receptivity (Fig. 1). Nevertheless, EP as an entity is critiqued due to methodological challenges in defining what constitutes an ‘optimal’ progesterone level^1,2^. Previous systematic reviews on EP have reported conflicting results^3–6^. Progesterone supplementation is used in the luteal phase of modified natural and medicated frozen embryo transfer (FET) cycles to ensure a sufficient hormonal environment. However, what constitute an adequate luteal phase progesterone level is also not well defined^7^. Current practice now involves blastocyst transfer; day 5 embryos are known to be more robust but studies evaluating the impact of progesterone monitoring do not differentiate day 3 versus day 5 transfers.

This review aims to investigate if progesterone levels at different phases of fresh and frozen ART cycles influence pregnancy outcomes, in particular, that on cleavage-stage versus blastocyst embryo transfers. The main outcome is live birth rate (LBR). Additional outcome measures are the ongoing pregnancy rate (OPR), clinical pregnancy rate (CPR) and miscarriage rate (MR).

## Methods

### Search strategy

**Fig. 2:**
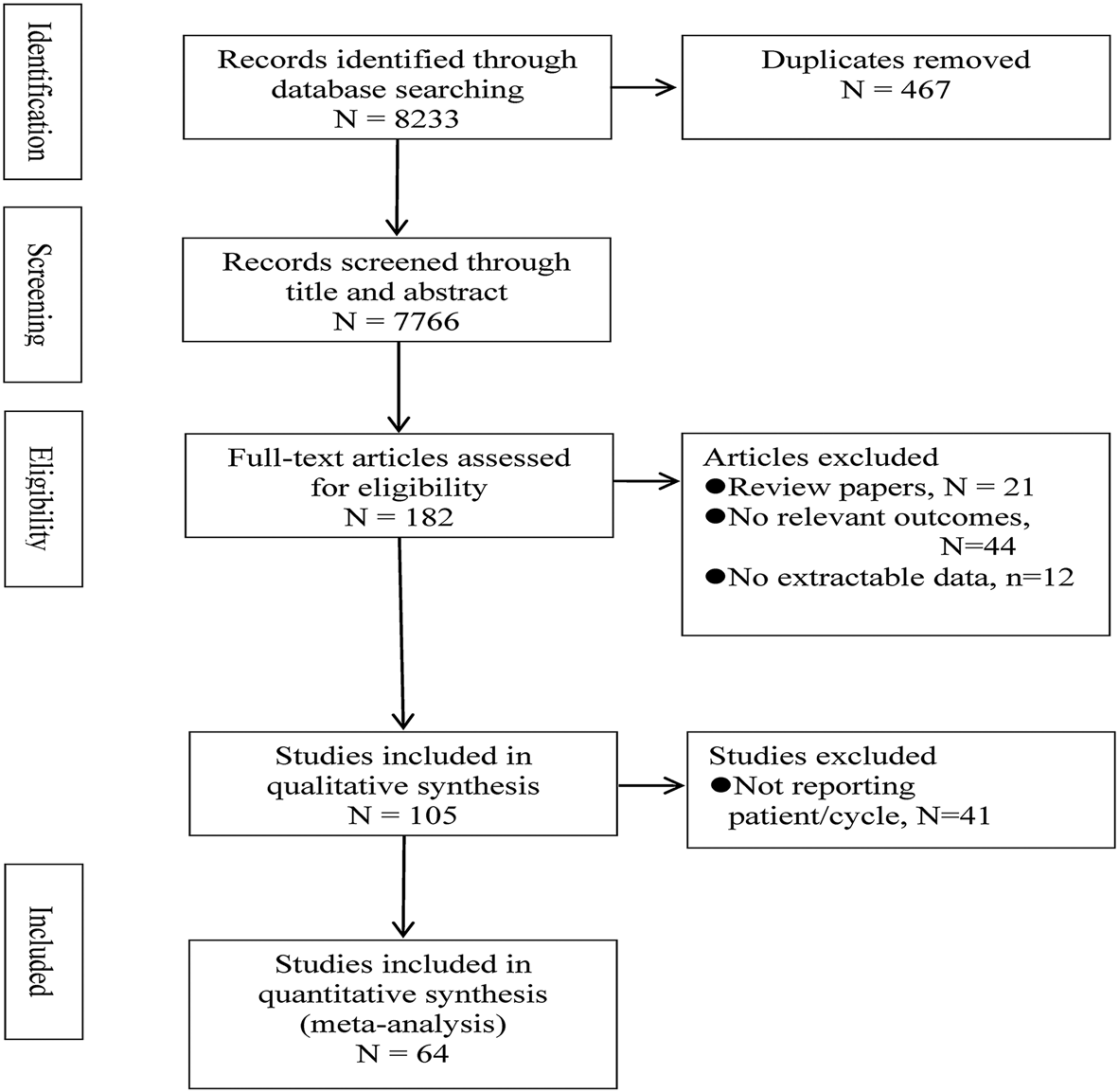

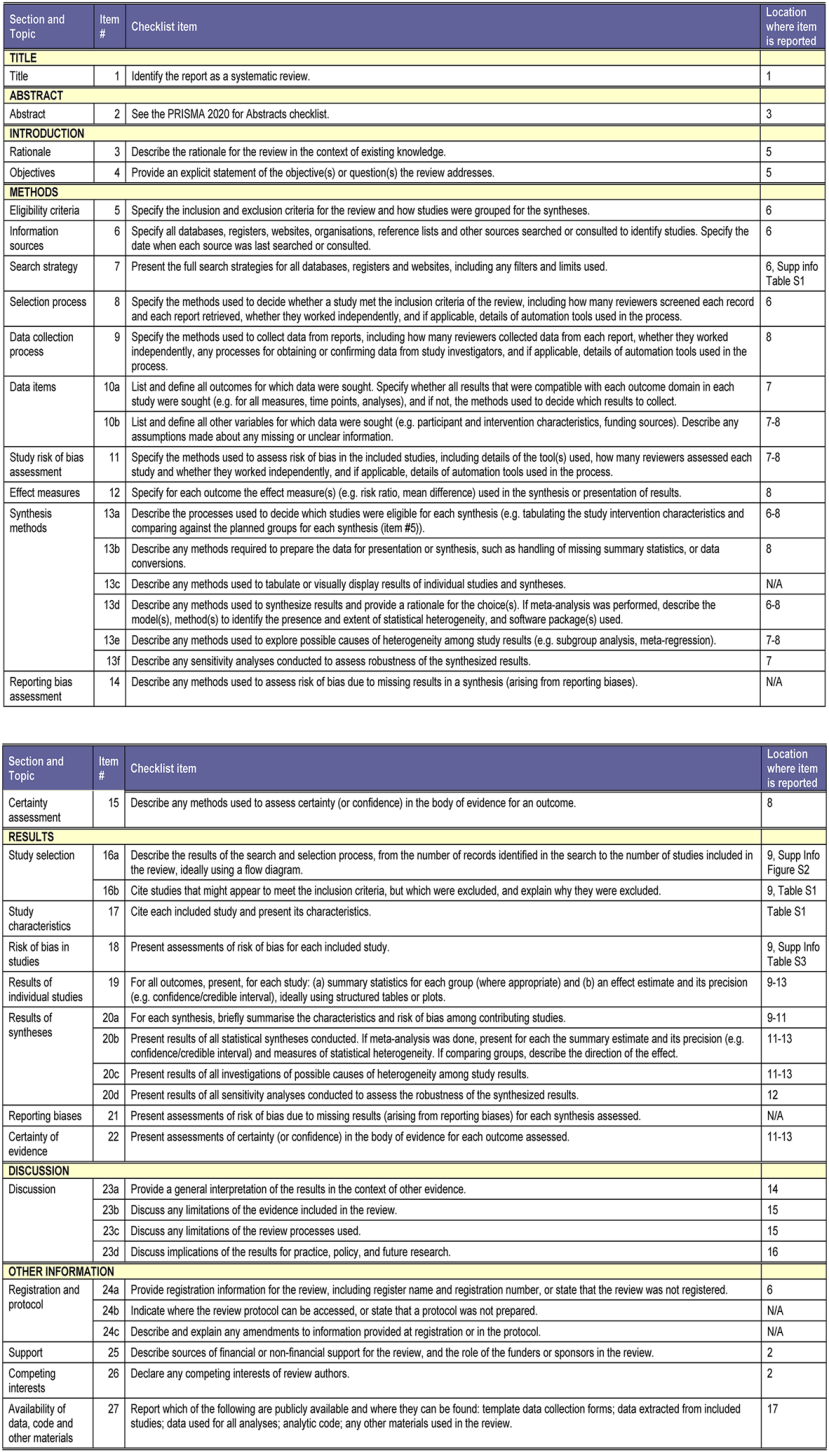
PRISMA flow diagram.

A systematic search was performed on all published studies in EMBASE, MEDLINE, CINAHL, PubMed, SCOPUS and Web of Science following PRISMA and the MOOSE guidelines (Fig. 2) by starting the search after the year 2000. The search from the year 2000 was chosen due to a change of practice in IVF with the introduction of GnRH antagonists. The study was registered with PROSPERO (registration ID CRD42022382423).

### Selection of studies

The titles and abstracts retrieved were initially screened by two reviewers independently (Y.C.L and M.H.) and the full texts that meet the predefined criteria were examined for compliance with the inclusion criteria. Studies eligible for inclusion were selected. In cases of duplicate publication, the most recent version was selected. Studies that specified reporting per woman data were reported to reduce confounding.

### Study protocol PICOS

#### Population

The inclusion criteria included (a) studies on fresh IVF/ICSI cycles or natural/modified natural/medicated FET cycles, (b) controlled ovarian stimulation (COS) with gonadotrophins and GnRH analogues in fresh cycle, or using trigger in modified natural FET cycle, or using hormonal replacement therapy in medicated FET cycle (c) the study provided extractable per woman data on pregnancy outcomes which included live birth rate (LBR), ongoing pregnancy rate (OPR), clinical pregnancy rate (CPR), miscarriage rate (MR) and (d) where serum progesterone was monitored.

The exclusion criteria included (a) any intervention that leads to cycle cancellation or freeze-all embryos in the follicular phase or further progesterone supplementation in the luteal phase of fresh and frozen embryo transfer cycles, (b) studies involving donor cycles, (c) studies without control groups and (d) studies providing per cycle data on pregnancy outcomes. Any intervention in the studies that influence the clinical decision and change the pregnancy outcome is excluded from the review.

#### Comparisons

We made the following comparisons:

(A) Fresh ovarian stimulation cycle with embryo transfer (ET)
  i. Basal follicular phase comparing EP versus non-elevated progesterone (NEP)
  ii. At ovulation trigger comparing EP versus NEP
  iii. At egg collection comparing EP versus NEP
  iv. Luteal phase comparing adequate versus inadequate progesterone level
(B) Frozen embryo transfer (FET) cycle
  i. Modified natural cycle FET (NC-FET) at trigger comparing EP versus NEP
  ii. NC-FET: comparing EP versus NEP on the day before ovulation
  iii. Luteal phase comparing adequate versus inadequate progesterone level
    - Natural cycle with or without progesterone supplementation
    - Medicated FET cycle

### Outcome measures

The primary outcome was LBR and the secondary outcomes were OPR, CPR and MR. The definitions for these outcomes were in accordance with the ICMART glossary^8^.

Comparative pregnancy outcomes were assessed based on the authors’ predefined progesterone threshold. In studies using multiple threshold ranges, the outcome data were dichotomized based on all the reported thresholds in the individual study. The conversion factor of 3.18 was used to convert units in nmol/l to units ng/ml.

We included results from published cohort or case–control studies (retrospective or prospective), and data from randomised control trials (RCT) where EP and NEP were analysed as subgroups. The data for EP and NEP groups in both arms of intervention were pooled together and analysed as cohort studies. Systematic reviews and meta-analyses were included for qualitative and quantitative data where appropriate. The studies were initially analysed together regardless of Day 3 or Day 5 embryos. We then performed subgroup analysis on the studies that measured either Day 3 or Day 5 embryos individually. We did not compare pregnancy outcomes between Day 3 and Day 5 embryos.

### Assessment of study quality and data extraction

The Newcastle Ottawa Scale (NOS) was used to determine bias in the non-randomised comparative cohort studies. Each study was judged based on eight items categorised into three domains: the study group selection, the comparability of the groups, and the ascertainment of the outcome of interest. Scores were represented with stars for each quality item and a maximum of nine stars awarded if they fulfilled all the quality items^9^. The Newcastle Ottawa Scale is derived to assess non-randomized controlled trials. We chose NOS as it is one of the most known scales for assessing quality and risk of bias in observational studies. It is easily adaptable and validated for case–control and long-term studies, although the authors acknowledge its drawbacks^10^.

Data were extracted by 2 independent reviewers (Y.C.L. and M.H.). Any disagreements were resolved by a third author (Y.C.). Data retrieved included study characteristics and their various outcomes data. Both reviewers counterchecked these extracted data repeatedly. Authors were contacted for further data through email. Data were extracted into RevMan5 for further analysis.

### Data analysis and assessment of heterogeneity

Data were extracted in 2 × 2 tables for dichotomous outcomes. The odds ratio (OR) for dichotomous outcomes with 95% CI for each study were estimated. The estimates were pooled using the DerSimonian and Laird random-effects model, which uses inverse variance weighting for random effects meta-analysis. The random effects model was chosen a priori to pool the results from individual studies given the increased clinical heterogeneity of the population assessed, the wide variation of thresholds adopted by studies, different responder types, different types of protocols, different stages of embryo development transfer and in fresh and frozen cycles with variable outcomes. Meta-analysis was not performed on single studies and studies where progesterone thresholds were too variable for meaningful meta-analysis. A p-value of < 0.05 is considered statistically significant.

We considered whether the clinical and methodological characteristics of the included studies were sufficiently similar for meta-analysis to provide a clinically meaningful summary. Statistical heterogeneity was assessed by the measure of the I^2^. Scores below 50% were considered to represent low or moderate heterogeneity^11^. The incorporation of a random-effects meta-analysis model involved an assumption that the effects being estimated in the different studies are not identical but follow some distribution.

### Rating quality of evidence and strength of evidence (GRADE)

The GRADE tool was used to assess the strength of evidence for significant outcomes. There were four categories of evidence quality based on the overall GRADE scores for each comparison as per the GRADE recommendations (high, moderate, low and very low)^12^.

### Ethics application

Ethics application was not required for this study.

## Results

The systematic search retrieved 7766 titles after removal of duplicates. One hundred eighty-two eligible studies had their full texts reviewed. One hundred five studies met our inclusion criteria and were included into the qualitative meta-analysis. A further forty-one studies that did not report per woman data were excluded, leaving a total of sixty-four eligible studies (N = 57,988 women) for quantitative meta-analysis. Study identification and selection process is shown in Fig. 2.

For fresh COS cycle, three studies reported progesterone monitoring during the start of the menstrual cycle, forty-three studies reported monitoring during the day of trigger, three studies reported monitoring during egg collection day and three studies reported monitoring progesterone during the luteal phase. For FET cycles, one study reported monitoring on the day of trigger in modified NC-FET, two studies reported progesterone monitoring in NC-FET on the day before ovulation and nine studies reported monitoring progesterone during luteal phase in natural cycle FET with and without progesterone supplementation and medicated HRT cycle (Table 1). Supplementary Table S2 shows assessment for bias using NOS.

**Table 1:** Tables of included studies.

Author/Year	Country	Study duration	Study design	Type of cycle	Total number (patient/cycle)	Threshold/reason for choosing	Day of ET	Conclusion
Fresh COS cycle—Basal follicular phase								
Hamdine et al., 2014	Netherlands	Mar’09 to Jul’11	Prospective	IVF/ICSI	158/158	P > 1.5 ng/ml/literature	Day 3	LBR, OPR and CPR NS
Mahapatro and Radhakrishan, 2017	India	Jan’13 to Mar’14	Retrospective	ICSI	151/151	P > 1.5 ng/ml/literature	Day 2–3	LBR and CPR NS
Mutlu et al., 2017	Turkey	Dec’14 and Feb’16	Prospective	ICSI	464/464	P ≥ 0.65 ng/ml/ROC analysis	Day 2,3 or 5	Similar OPR and CPR
Fresh COS cycle—Pre trigger								
Bosch et al., 2003	Spain	NA	Prospective	IVF/ICSI	81/81	P > 1.2 ng/ml/ROC analysis	Day 3	CPR ↓
Martinez et al., 2003	Spain	Jul’2 to Jan’03	Retrospective	IVF/ICSI	377/377	P > 0.9 ng/ml/ROC analysis	Day 2–3	CPR and MR NS
Anderson et al., 2006	Belgium	Feb’04 to Dec’04	RCT	IVF	731/731	P > 4 nmol/L (1.25 ng/ml)/literature	Day 3	COC ↑, OPR ↓
Seow KM et al., 2007	Taiwan	Jan’03 to Jan’05	Prospective	IVF/ICSI	95/95	P ≥ 1.2 ng/ml/literature	Day 2–3	CPR NS
Lee F et al., 2008	China	Mar’03 to Apr’07	Retrospective	IVF/ICSI	223/223	P > 2.0 ng/ml/arbitrary	Day 2–3	CPR ↓
Li R et al., 2008	China	Jul’06 to Dec’06	Prospective	IVF/ICSI	251/251	P > 3.97 nmol/L (1.25 ng/ml)/sensitivity–specificity analysis	Day 3	CPR ↓ in fresh cycle, CPR NS in FET
Kiliçdag et al., 2009	Turkey	Oct’04 to May’08	Retrospective	ICSI	1045/1045	P > 1.1 ng/ml/sensitivity–specificity analysis	Day 3	LBR, OPR and CPR ↓
Papanikolaou et al., 2009	Belgium	May’04 to Feb’05	Prospective	IVF/ICSI	482/482	P > 1.5 ng/ml/literature	Day 3 or 5	CPR ↓ in D3, similar CPR in D5
Rezaee et al., 2009	Iran	1 year (2009)	Prospective	Fresh cycle	38/38	P > 1.2 ng/ml/literature	Day 2	CPR ↑ but NS
Seow KM et al., 2010	Taiwan	Jun’04 to Jun’07	Prospective	IVF/ICSI	233/233	P > 1.2 ng/ml/ROC analysis	Day 3	CPR ↓
Elgindy, 2011	Egypt	Aug’08 to Jun’10	Prospective	ICSI	240/240	P > 1.5 ng/ml/ROC analysis	Day 3 or 5	CPR ↓ in Day 3 embryo, CPR NS in day 5 embryo
Lahoud et al., 2011	Australia	Jan’03 to Dec’03	Retrospective	IVF/ICSI	582/582	P ≥ 1.7 ng/ml/arbitrary	Day 2,3 or 5	CPR and MR NS, LBR ↓ in fresh cycle, similar LBR, CPR and MR in FET
Yding Anderson et al., 2011	Denmark	Aug’03 to Nov’04	Secondary data analysis from prospective RCT	IVF/ICSI	475/475	P > 1.25 ng/ml/arbitrary	NA	Similar CPR
Huang R et al., 2012	China	Jan’02, to Dec’07	Retrospective	IVF/ICSI	2566/2566	P > 1.2 ng/ml/arbitrary	Day 3	LBR ↓
Kyrou et al., 2012	Belgium	Oct’07 to Dec’08	Prospective	IVF/ICSI	207/207	P > 1.5 ng/ml/literature	NA	CPR ↓
Papanikolaou et al., 2012	Greece	Aug’07 to Dec’09	RCT	IVF/ICSI	190/190	P > 1.5 ng/ml/literature	Day 2,3 or 5	LBR ↓
Peng C et al., 2012	China	Jun’08 to Feb’10	Retrospective	IVF	180/180	P ≥ 1.2 ng/ml/literature	Day 3	CPR NS
Ochsenkuhn et al., 2012	Germany	Jan’06 to Jan’11	Retrospective	IVF/ICSI	2555/2555	P > 1.5 ng/ml/literature	Day 5	LBR ↓
Wu Z et al., 2012	China	Apr’08 to Apr’09	Retrospective	IVF/ICSI	2921/2921	P ≥ 1.05 ng/ml/literature	Day 3	LBR and CPR ↓ in fresh cycles, CPR NS in FET
Corti et al., 2013	Italy	Jan’12 to Dec’12	Retrospective	IVF/ICSI	204/204	P > 1.5 ng/ml/literature	Day 5	OPR and CPR ↓
Griesinger et al., 2013	Germany	NA	Pooled analysis of 6 RCTs	IVF/ICSI	1866/1866	P > 1.5 ng/ml/literature	Day 3	OPR ↓
Orvieto et al., 2013	Israel	10-year period	Retrospective	IVF	2244/2244	P > 1.5 ng/ml/literature	NA	CPR ↓
Papaleo et al., 2014	Italy	Aug’11 and Jan’12	Retrospective	IVF/ICSI	303/303	P > 1.35 ng/ml/ROC analysis	Day 3	CPR ↓
Acet et al., 2015	Turkey	Nov’12 to Feb’14	Retrospective	IVF/ICSI	101/101	P ≥ 1.3 ng/ml/literature	Day 5	similar LBR, CPR and MR
Huang P et al., 2015	Taiwan	Jan’10 to Dec’12	Retrospective	IVF/ICSI	599/599	P > 1.5 ng/ml/literature	Day 2,3 or 5	LBR and CPR ↑
Huang Y et al., 2015	China	Jan’10 to Oct’14	Retrospective	IVF/ICSI	12,010/12,010	Day 3, P ≥ 1.5 ng/ml; Day 5 P ≥ 1.75 ng/ml/arbitrary	Day 3 or 5	CPR ↓
Koo et al., 2015	Korea	May’12 to Jul’13	Prospective	IVF/ICSI	200/200	P > 0.9 ng/ml/arbitrary	Day 3	CPR ↓
Singh et al., 2015	India	Jan’12 to Jul’14	Retrospective	IVF/ICSI	681/681	P > 1.0 ng/ml/ROC analysis	Day 3 or 5	CPR ↓
Tsai Y et al., 2015	Taiwan	Jan’00 to Dec’12	Retrospective	IVF/ICSI	1508/1508	P > 1.94 ng/ml/ROC analysis	Day 3 or 5	LBR, OPR and CPR ↓
Demir et al., 2016	Turkey	Jan’12 to Jun’14	Prospective	ICSI	201/201	P > 2 ng/ml/arbitrary	Day 3 or 5	CPR NS
Healy et al., 2016	USA	2011 to 2013	Retrospective	IVF/ICSI and FET	608/608	P ≥ 2 ng/ml/literature	Day 3 or 5	LBR ↓ in fresh cycle, LBR similar in FET
Ashmita et al., 2018	India	Jan’16 to Dec’16	Prospective	IVF/ICSI	235/235	P > 1.5 ng/ml/arbitrary	Day 3	CPR ↓
Simon et al., 2019	France	Sep’12 and Jul’17	Retrospective	IVF/ICSI	1399/1399	P > 1.10 ng/ml/arbitrary	Day 2–3	CPR ↓
Wu et al., 2019	China	Jan’08 to Mar’11	Retrospective	IVF/ICSI	2351/2351	P > 1.0 ng/ml in low ovarian response/arbitrary; P ≥ 2.0 ng/ml in intermediate ovarian response/arbitrary	Day 3	LBR and CPR↓ in low and intermediate ovarian response
Lee C et al., 2020	Taiwan	Feb’11 to Oct’16	Retrospective	IVF/ICSI	337/337	P > 1.5 ng/ml/literature	Day 3	LBR ↓, CPR and MR NS
Yu Y et al., 2020	China	2013 to 2017	Secondary analysis of 3 RCTs	IVF/ICSI and natural cycle/HRT FET	5137/5137	P > 1.14 ng/ml/ROC analysis	Day 3 or 5	LBR and CPR in FET ↑ than fresh cycle
Benmachiche et al., 2021	Denmark	2014 to 2016	Retrospective	IVF/ICSI	328/328	P > 1.3 ng/ml/arbitrary	Day 2–3	CPR and LBR NS
Mahran et al., 2021	Egypt	Oct’16 to May’18	Prospective	IVF/ICSI	200/200	P > 1 ng/ml/ROC analysis	Day 3 or 5	CPR NS
Mirta et al., 2021	India	Jan’13 to Jun’16	Retrospective	IVF/ICSI	273/273	P > 1.5 ng/ml/literature	Day 2–3 or Day5-6	CPR and MR NS
Yang et al., 2021	China	Jun’13 and Sep’20	Retrospective	IVF/ICSI	1254/1254	P ≥ 0.9 ng/ml/ROC analysis	Day 3 or 5	LBR, CPR and MR NS
Jiang W et al., 2022	China	Jan’16 to Oct’16	Retrospective	IVF/ICSI	2550/2550	P > 1.5 ng/ml/literature	Day 5	LBR and CPR ↓
Kong N et al., 2022	China	Jan’18 to Dec’20	Retrospective	IVF	1951/1951	P > 1.5 ng/ml/literature	Day 3 or 5	LBR, CPR and MR NS
Zhao et al., 2022	China	Jan’20 to Apr’21	Retrospective	IVF/ICSI	455/455	P ≥ 1.0 ng/ml/arbitrary	Day 3	CPR ↓
Fresh COS cycle—Day of transvaginal oocyte retrieval								
Niu Z et al., 2008	China	May’05 to May’07	NA	ICSI	289/289	P > 11.7 ng/ml sensitivity–specificity analysis	Day 3	OPR and CPR NS
Nayak et al., 2014	USA	Feb’10 and May’12	Prospective	IVF/ICSI	186/186	P > 12 ng/ml/arbitrary	Day 3	CPR ↓, MR NS
Tulic et al., 2020	Serbia	Jan’15 to Dec’15	Prospective	IVF/ICSI	164/164	P ≥ 2 ng/ml/ROC analysis	Day 2–3	LBR ↓
Fresh COS cycle—luteal phase								
Kim et al., 2017	S. Korea	NA	Prospective	IVF-ET	148/148	P > 25.2 ng/ml (ROC analysis)	Day 3	OPR ↑, MR ↓
Thomsen et al., 2018	Denmark	May’14 to Jun’17	Prospective	IVF/ICSI-ET	602/602 Early luteal phase—432 Mid-luteal phase—170	Early luteal phase - P < 18.9 ng/ml; P = 18.9 -31.4 ng/ml; P = 31.8–125.8 ng/ml; P > 125.8 ng/ml Mid-luteal phase - P < 47.2 ng/ml; P = 47.2–78.6 ng/ml; P = 78.6–125.8 ng/ml; P > 125.8 ng/ml	Day 2,3 or 5	Optimal chance of pregnancy P = 60–100 nmol/L (early luteal phase) and P = 150- 250 nmol/L (mid-luteal phase)
Netter et al., 2019	France	Jul’17 and Jun’18	Retrospective	IVF/ICSI-ET	242/242	P < 36.1 ng/ml P = 36.1–79.2 ng/ml P > 79.2 ng/ml	Day 2–3	LBR ↑ when P > 252 nmol/L
FET cycle—day of trigger in modified FET cycle								
Groenewoud et al., 2017	Netherlands	Part of “ANTARTICA” trial	Secondary analysis of RCT	Modified NC FET	271/271	P > 1.47 ng/ml/ROC analysis	Day 3 or 5	LBR NS
FET cycle—at day before ovulation								
Lee VC et al., 2014	China	Jan’06 and Dec’11	Retrospective	NC FET	610/610	P > 1.57 ng/ml arbitrary	Day 3	OPR and CPR NS
Wu D et al., 2022	China	Jan’18 to Apr’20	Retrospective	NC FET	1159/1159	P > 1.0 ng/ml	Day 3 or 5	LBR NS, CPR ↑, MR NS in day 3 LBR, CPR and MR NS in day 5
FET cycle—luteal phase								
Akaeda et al., 2019	Japan	Sep’10 to Sep’15	Retrospective	HRT FET	123/123	P < 5 ng/ml; P = 5–9.9 ng/ml P = 10–14.9 ng/ml; P ≥ 15 ng/ml	Day 2,3 or 5	Optimal chance of pregnancy P = 5-15 ng/ml
Boynukalin et al., 2019	Turkey	Mar’18 to Aug’18	Prospective	HRT FET	168/168	P < 13.6 ng/ml; P = 13.6–24.3 ng/ml P = 24.4–53.2 ng/ml; P > 53.2 ng/ml	Day 5	OPR ↑, MR ↓when P > 13.6 ng/ml
Alsbjerg et al., 2020	Denmark	Mar’18 and Apr’19	Prospective	HRT FET	239/239	P < 8.8 ng/ml; P = 8.8–14.2 ng/ml P > 14.2 ng/ml	Day 5–6	OPR, MR NS
Liu and Wu, 2020	China	Jan’15 to Dec’18	Retrospective	HRT FET	856/262 (only IM group)	P > 13.15 ng/ml (arbitrary)	Day 2–3	LBR NS
Polat et al., 2020	Turkey	Oct’17 to Oct’19	Retrospective	HRT FET	475/475	PV only: P < 8.75 ng/ml; P = 8.76–12.94 ng/ml; P = 12.95–20.42 ng/ml; P > 20.42 ng/ml PV + IM: P < 11.75 ng/ml; P = 11.76–19.86 ng/ml; P = 19.87–31.79 ng/ml; P > 31.79 ng/ml	Day 5–6	No correlation between serum P level and OPR, CPR or MR
Shiba et al., 2021	Japan	Dec’16 to Dec’17	Secondary analysis of RCT	HRT FET	235/235	P < 7.8 ng/ml; P = 7.8–10.8 ng/ml P = 10.8–13.7 ng/ml; P > 13.7 ng/ml	Day 3 or 5	LBR, CPR and MR NS
Alyasin et al., 2021	Iran	Feb’19 and Feb’20	Prospective	HRT FET	258/258	P < 19 ng/ml; P = 19–29 ng/ml P = 29–49 ng/ml; P > 49 ng/ml	Day 5	LBR and CPR significantly lower in 4th quartile, MR NS
Maignien et al., 2022	France	Jan’19 and Mar’20	Retrospective	HRT FET	915/915	< 9.8 ng/ml (previous study)	Day 5	LBR ↓, CPR NS and MR ↑
Melo et al., 2022	UK	January 2020	Prospective	NC FET/HRT FET	402/402	< 7.8 ng/ml (10th centile)	Day 5	LBR↑, CPR ↑ and MR ↓ when P4 increasing trend

Table showing characteristics of included studies with their progesterone threshold/range and the summary of pregnancy outcomes reported in each studies.

*COS* controlled ovarian stimulation, *CPR* clinical pregnancy rate, *ET* embryo transfer, *EP* elevated progesterone, *FET* frozen embryo transfer, *HRT* hormone replacement therapy, *ICSI* intracytoplasmic sperm injection, *IM* intramuscular, *IVF* in vitro fertilization, *LBR* live birth rate, *MR* miscarriage rate, *NC* natural cycle, *NEP* non-elevated progesterone, *NS* non-significant, *OPR* ongoing pregnancy rate, *P/P4* progesterone, *RCT* randomized-controlled trial, *TVOR* transvaginal oocyte retrieval.

### Study characteristics

#### Fresh ovarian stimulation cycle with ET

##### i. At basal follicular phase

Three studies^3,13,14^ reported progesterone monitoring in this category. Serum progesterone was measured on day 2 of the menstrual cycle. Two thresholds were identified, P > 0.65 ng/ml and P > 1.5 ng/ml. Two studies reported using D3 embryos^3,13^ and one study reported both D3 and D5 embryos^14^ (Table 1).

##### ii. At day of ovulation trigger

Forty-three studies had progesterone monitoring in this category^15–57^. The trigger used were HCG or agonist trigger. The progesterone threshold ranged from 0.9 to 2.0 ng/ml. Twenty-one studies reported using D3 embryos^15–21,23,24,28,31,33,35,37,41,46–49,51,57^, four studies reported using D5 embryos^32,34,38,55^, eighteen studies reported using both D3 and D5 embryos^22,25,26,30,39,40,42–45,50,52–54,56^ and three studies did not specify the stages of embryo used^27,29,36^ (Table 1).

##### iii. At egg collection

Three studies reported progesterone monitoring in this category^58–60^. The progesterone threshold level used ranged from 2 to 12 ng/ml. All three studies reported using D3 embryos (Table 1).

##### iv. At luteal phase

Three studies reported progesterone monitoring in this category^61–63^. The timing of serum progesterone measurements varied widely from the day of ET (two studies)^62,63^ and after ET (one study)^61^. Two studies used vaginal suppositories^61,62^ and one study used oral progesterone^63^. One study^61^ reported a single progesterone threshold level (< 25.2 ng/ml) and the other two studies^62,63^ reported progesterone level in ranges (< 115 nmol/L, 115–252 nmol/L and > 252 nmol/L^63^; 10th/50th/90th percentile for early luteal phase and 25th/50th/75th percentile in mid luteal phase^62^ ). Two studies reported the use of D3 embryos^61,63^ and one study reported using both D3 and D5 embryos^62^ (Table 1).

#### FET cycle

##### i. At ovulation trigger in modified NC-FET cycle

One study reported EP at ovulation trigger^64^. The progesterone threshold level was > 1.47 ng/ml (Table 1).

##### ii. Before ovulation in NC-FET cycle

Two studies reported EP in this category^65,66^, and ovulation was determined by either monitoring of LH surge or when the collapse of the dominant follicle was observed during transvaginal scan. The progesterone threshold levels were > 1.0 ng/ml and > 1.57 ng/ml. One study reported the use of D3 embryos^65^ and the other study reported using both D3 and D5 embryos^66^ (Table 1).

##### iii. At luteal phase

Nine studies reported progesterone monitoring in this category^67–75^. All the studies apart from one^75^ were medicated FET cycles. No studies reported progesterone monitoring in natural FET cycle with or without progesterone supplementation. Melo et al.^75^ included women from natural, and medicated FET cycles. The timing of serum progesterone measurements varied widely from the day of ET (seven studies)^67,68,71–75^ and after ET (two studies)^69,70^. Three studies used vaginal suppositories^71,72,74^, two studies used intramuscular injections^68,70^ and four studies used a combination of progesterone support^69,71,73,75^. Three studies^70,74,75^ reported single progesterone threshold level (< 7.8 ng/ml, < 9.8 ng/ml and < 13.15 ng/ml) and the remaining six studies^67–69,71–73^ reported progesterone value according to quartiles or percentiles. One study reported the use of D3 embryos^70^, six studies reported using D5 embryos^68,69,71,73–75^ and two studies reported using both D3 and D5 embryos^67,72^ (Table 1).

#### Outcomes: fresh ovarian stimulation cycle with ET

##### A. At basal follicular phase

**Fig. 3:**
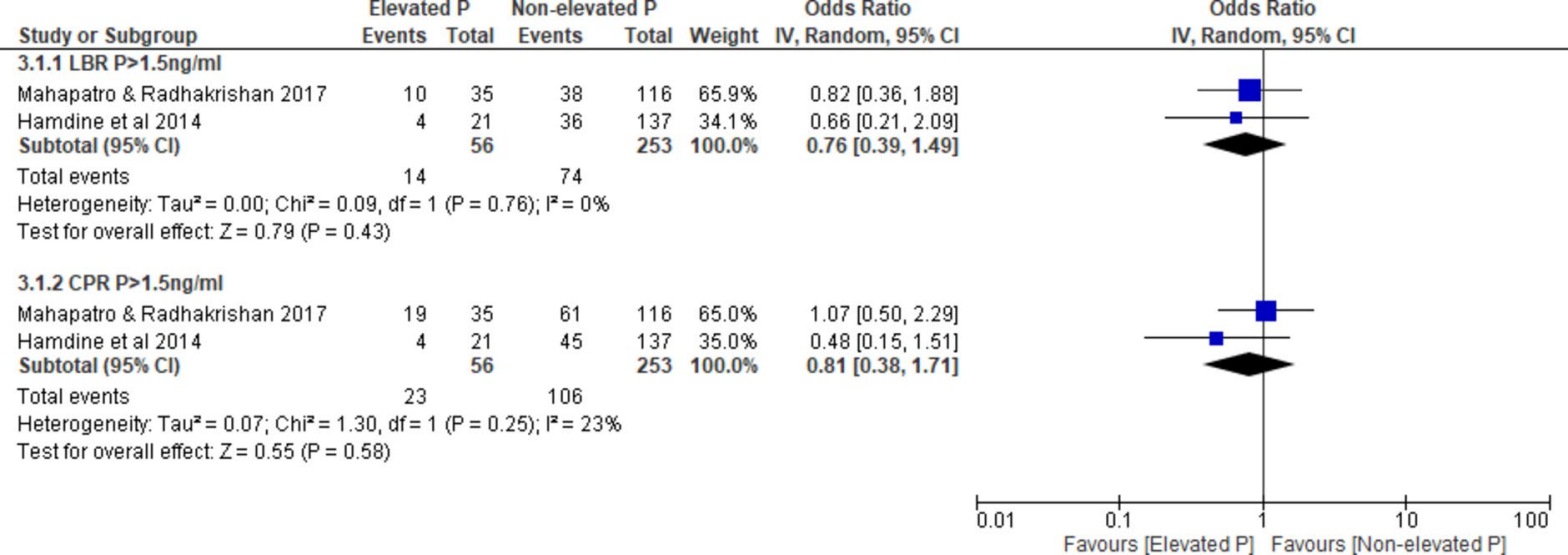
EP vs NEP at basal follicular phase, outcome: LBR and CPR. Forest plot of comparison between EP group and NEP group on LBR and CPR at basal follicular phase in fresh COS cycle. *COS* controlled ovarian stimulation, *CPR* clinical pregnancy rate, *EP* elevated progesterone, *LBR* live birth rate, *NEP* non-elevated progesterone.

There was no difference in LBR in the EP compared to the NEP at threshold level > 1.5 ng/ml, (OR 0.76, 95% CI 0.39–1.49, I^2^ = 0%, 2 studies, N = 309, very low quality) (Fig. 3).

Three studies^3,13,14^ reported CPR over two different threshold levels (> 0.65 ng/ml and > 1.5 ng/ml). There was no difference in CPR in the EP compared to the NEP (P > 0.65 ng/ml, OR 1.41, 95% CI 0.93–2.13, 1 study, N = 464; P > 1.5 ng/ml, OR 0.81, 95% CI 0.38-1.71, I^2^ = 23%, 2 studies, N = 309, very low quality) (Fig. 3).

We were unable to meta-analyse OPR and MR in a meaningful way as they are single studies. Data from single studies were summarised in Supplementary Table S3.

##### B. At day of ovulation trigger

**Fig. 4:**
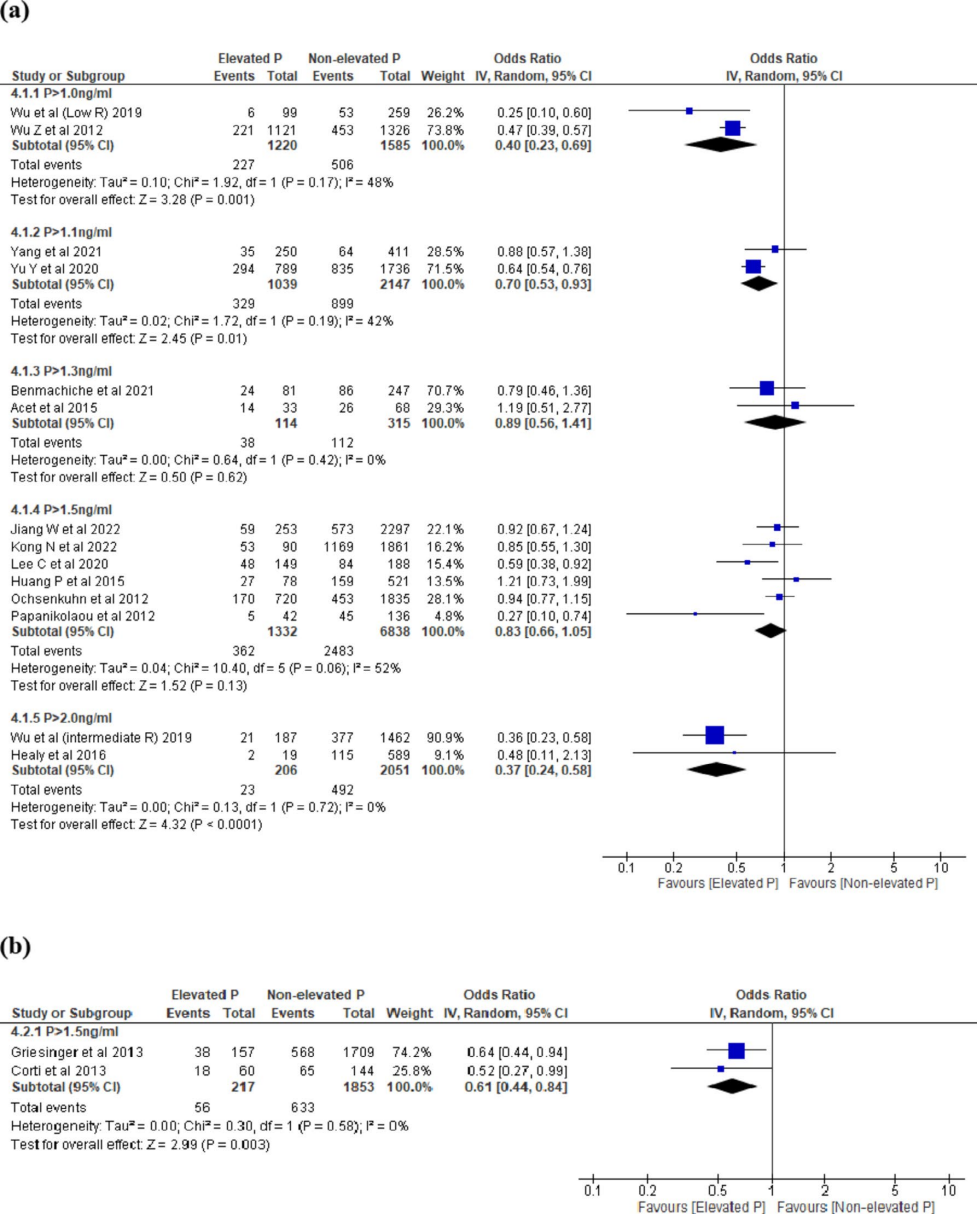

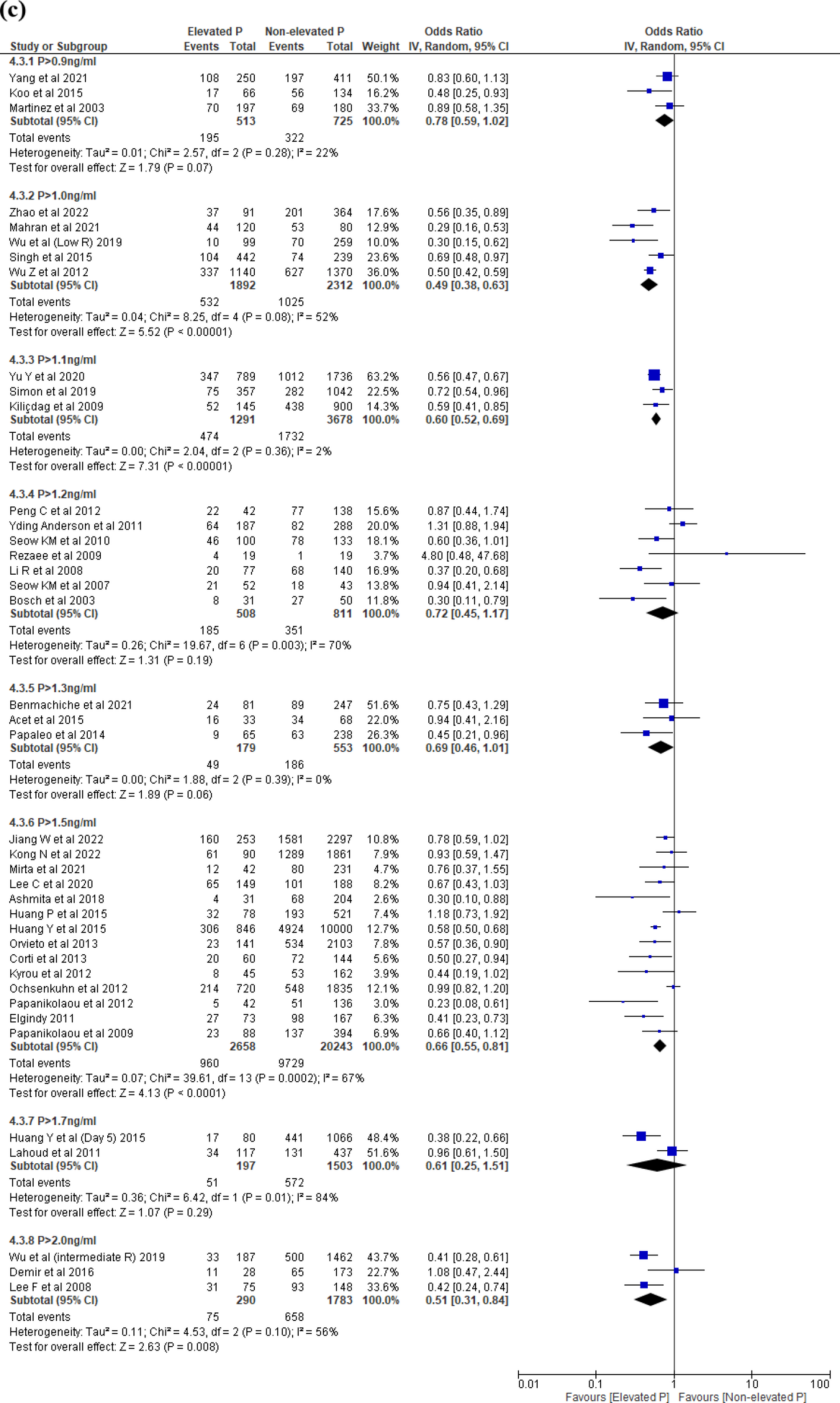

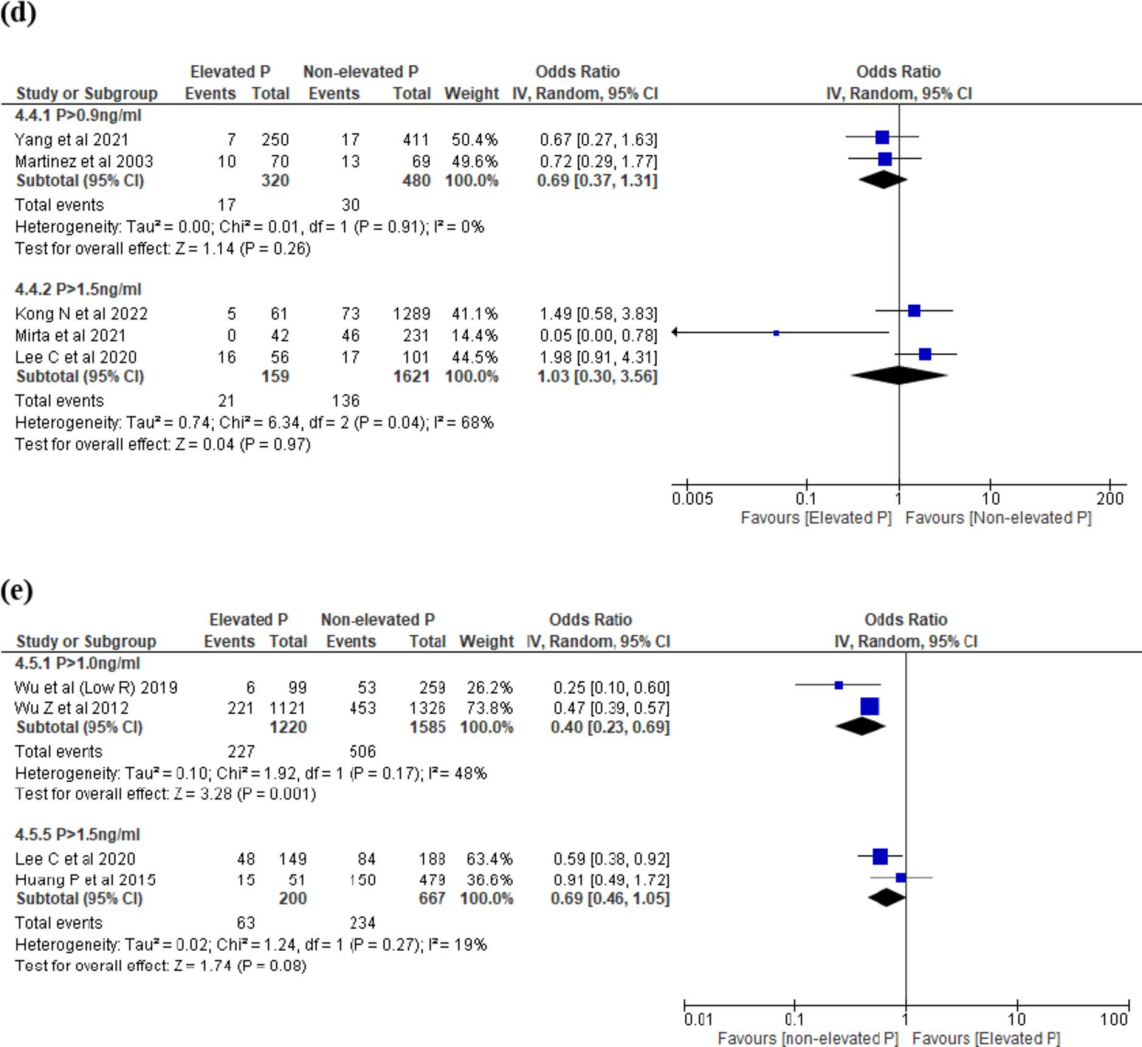

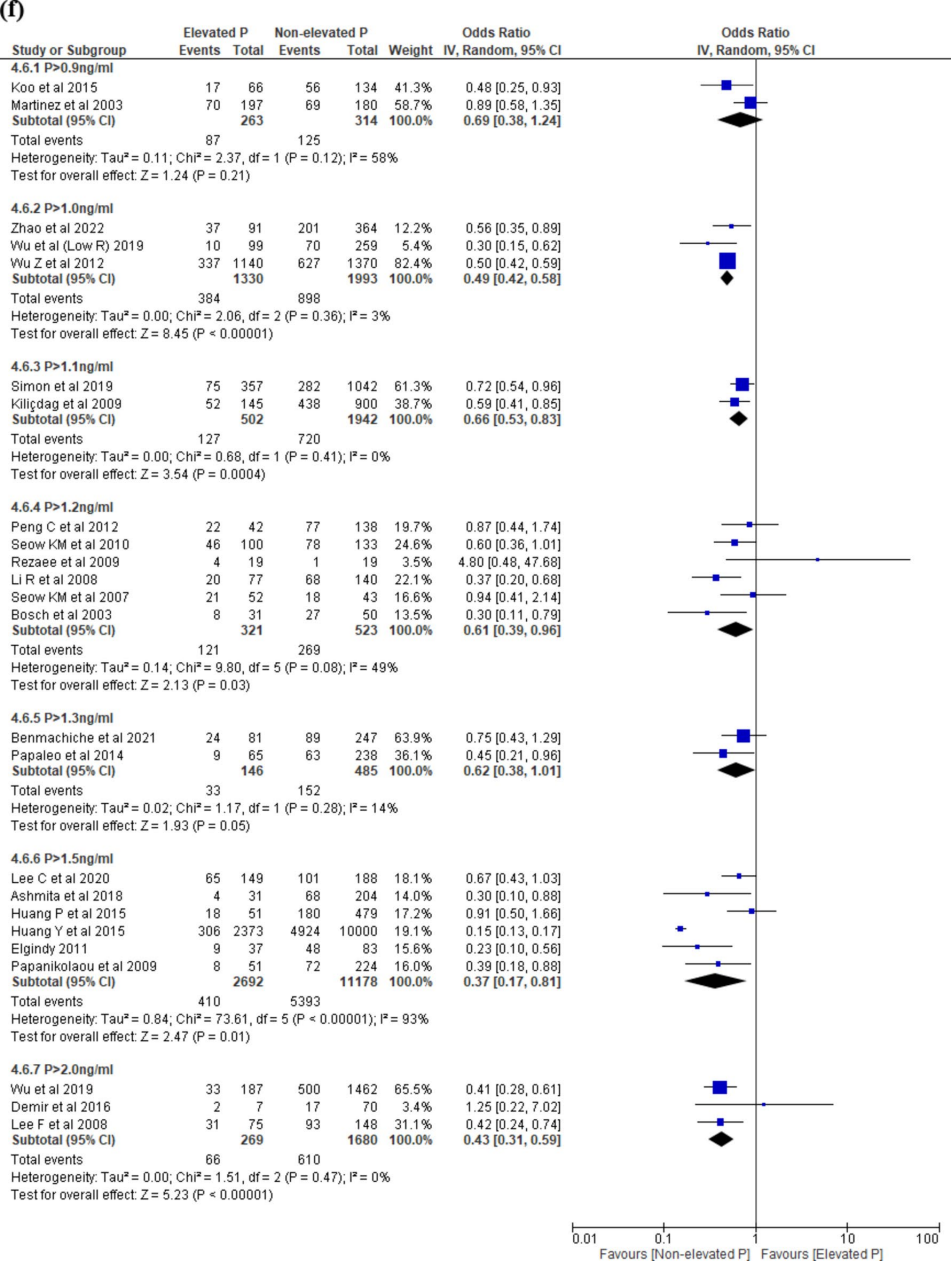

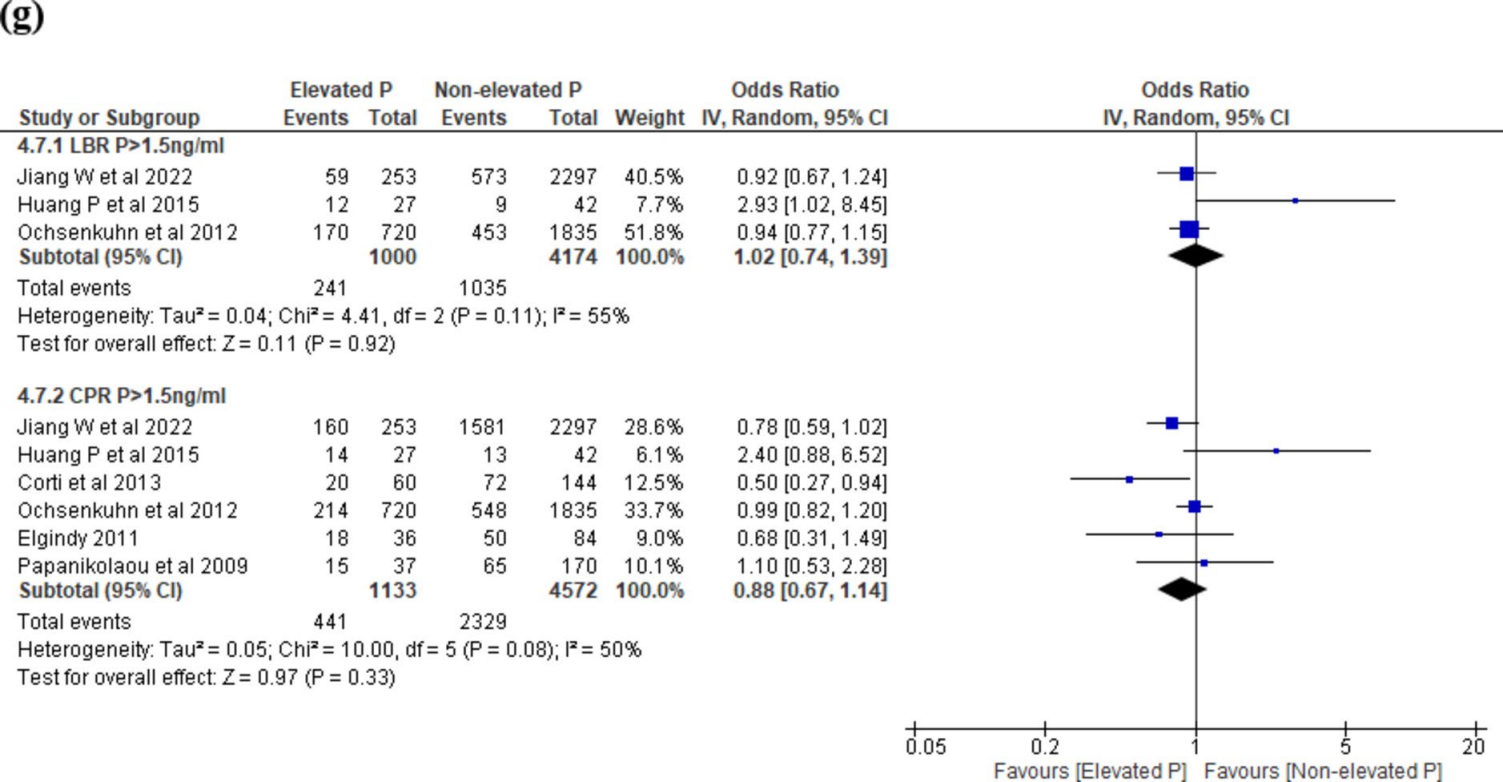
(**a**) EP vs NEP at ovulation trigger, outcome: LBR. (**b**) EP vs NEP at ovulation trigger, outcome: OPR. (**c**) EP vs NEP at ovulation trigger, outcome: CPR. (**d**) EP vs NEP at day of ovulation trigger, outcome: MR. (**a**–**d**) Forest plot of comparison between EP group and NEP group on LBR, OPR, CPR and MR at day of ovulation trigger in fresh COS cycle. *COS* controlled ovarian stimulation, *CPR* clinical pregnancy rate, *EP* elevated progesterone, *Intermediate R* intermediate ovarian response, *LBR* live birth rate, *Low R* low ovarian response, *MR* miscarriage rate, *NEP* non-elevated progesterone, *OPR* ongoing pregnancy rate. (**e**) EP vs NEP at day of ovulation trigger (Day 3 embryo), outcome: LBR. (**f**) EP vs NEP at day of ovulation trigger (Day 3 embryo), outcome: CPR. (**e**,**f**) Subgroup analysis on Day 3 embryo, Forest plot of comparison between EP group and NEP group on LBR and CPR at day of ovulation trigger in fresh COS cycle. *COS* controlled ovarian stimulation, *CPR* clinical pregnancy rate, *EP* elevated progesterone, *LBR* live birth rate, *Low R* low responder, *NEP* non-elevated progesterone. (**g**) EP vs NEP at day of ovulation trigger (Day 5 embryo), outcome: LBR and CPR. Subgroup analysis on Day 5 embryo, Forest plot of comparison between EP group and NEP group on LBR and CPR at day of ovulation trigger in fresh COS cycle. *COS* controlled ovarian stimulation, *CPR* clinical pregnancy rate, *EP* elevated progesterone, *LBR* live birth rate, *NEP* non-elevated progesterone.

Seventeen studies^21,26,28,30,32,33,38,39,43,45,48–51,54–56^ reported LBR. The threshold levels ranged between > 0.9 ng/ml to > 2.0 ng/ml. EP on the day of trigger was associated with decreased LBR across 3 threshold levels (P > 1.0 ng/ml, OR 0.40, 95% CI 0.23–0.69, I^2^ = 48%, 2 studies, N = 2805, very low quality; P > 1.1 ng/ml: OR 0.70, 95% CI 0.53–0.93, I^2^ = 42%, 2 studies, N = 3186, very low quality; P > 2.0 ng/ml: OR 0.37, 95% CI 0.24-0.58, I^2^ = 0%, 2 studies, N = 2257, very low quality) and no difference in LBR at 2 thresholds (P > 1.3 ng/ml, OR 0.89, 95% CI 0.56–1.41, I^2^ = 0%, 2 studies, N = 429, very low quality; P > 1.5 ng/ml: OR 0.83, 95% CI 0.66-1.05, I^2^ = 52%, 6 studies, N = 8170, very low quality) (Fig. 4a).

Five studies^17,21,34,35,43^ reported OPR. The threshold levels ranged between > 1.1 to > 1.9 ng/ml. Elevated progesterone level on the day of trigger was associated with decreased OPR in P > 1.5 ng/ml compared to those with NEP (OR 0.61, 95% CI 0.44–0.84, I^2^ = 0%, 2 studies, N = 2070, very low quality) (Fig. 4b).

Forty studies^15,16,18–27,29–34,36–44,46–57^ reported CPR. The threshold levels ranged between > 0.9 to > 2.0 ng/ml. EP on the day of trigger was associated with decreased CPR across 4 threshold levels: P > 1.0 ng/ml; P > 1.1 ng/ml; P > 1.5 ng/ml and P > 2.0 ng/ml (Fig. 4c) and no difference in CPR over 4 thresholds: P > 0.9 ng/ml; P > 1.2 ng/ml; P > 1.3 ng/ml; P > 1.7 ng/ml.

Nine studies^16,26,33,38,49,50,53,54,56^ reported MR. The threshold levels ranged between > 0.9 to > 1.7 ng/ml. There was no difference in MR in EP compared to NEP across all threshold levels: P > 0.9 ng/ml and P > 1.5 ng/ml (Fig. 4d). Data from single studies were summarised in Supplementary Table S3.

#### Subgroup analysis on Day 3 embryo at ovulation trigger

When we analysed studies which reported on only D3 embryos, there was a decreased LBR at threshold level > 1.0 ng/ml (OR 0.40, 95% CI 0.23–0.69, I^2^ = 48%, 2 studies, N = 2805, low quality) and no difference in LBR at > 1.5 ng/ml (OR 0.69, 95% CI 0.46–1.05, I^2^ = 19%, 2 studies, N = 867, low quality) (Fig. 4e). There was a decreased CPR at threshold levels (P > 1.0 ng/ml; OR 0.49, 95% CI 0.42–0.58, I^2^ = 3%, 3 studies, N = 3323, very low quality; P > 1.1 ng/ml; OR 0.66, 95% CI 0.53–0.83, I^2^ = 0%, 2 studies, N = 2444, low quality; P > 1.2 ng/ml; OR 0.61, 95% CI 0.39–0.96, I^2^ = 49%, 6 studies, N = 844, very low quality; P > 1.5 ng/ml; OR 0.37, 95% CI 0.17–0.81, I^2^ = 93%, 6 studies, N = 13,870, moderate quality; P > 2.0 ng/ml; OR 0.43, 95% CI 0.31–0.59, I^2^ = 0%, 3 studies, N = 1949, very low quality) (Fig. 4f) except at threshold levels > 0.9 ng/ml and > 1.3 ng/ml.

#### Subgroup analysis on Day 5 embryo at ovulation trigger

When we analysed studies which reported on only D5 embryos, there was no difference in LBR (P > 1.5 ng/ml; OR 1.02, 95% CI 0.74–1.39, I^2^ = 55%, 3 studies, N = 5174, very low quality) and CPR (P > 1.5 ng/ml; OR 0.88, 95% CI 0.67–1.14, I^2^ = 50%, 6 studies, N = 5705, very low quality) between EP and NEP groups (Fig. 4g).

##### C. At egg collection

One study^60^ reported LBR at threshold level > 2 ng/ml; one study^58^ reported OPR at threshold level > 11.7 ng/ml; three studies^58–60^ reported CPR at different threshold levels > 2 ng/ml, > 11.7 ng/ml and > 12 ng/ml; two studies^59,60^ reported MR at threshold levels > 2 ng/ml and > 12 ng/ml. Data from single studies were summarised in Supplementary Table S3.

##### D. Luteal phase

**Fig. 5:**
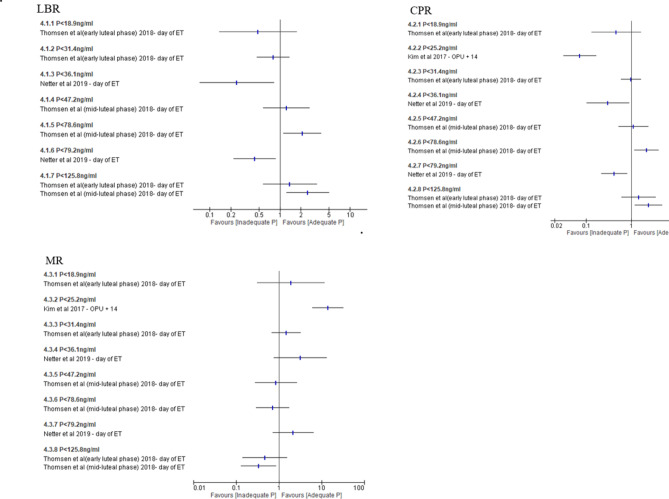
Inadequate vs adequate P during luteal phase of fresh COS cycle; outcome: LBR, CPR and MR. Forest plot of comparison on single studies between adequate progesterone group and inadequate progesterone group on LBR, CPR and MR during luteal phase in fresh COS cycle. *CPR* clinical pregnancy rate, *LBR* live birth rate, *MR* miscarriage rate, *P* progesterone.

Two studies^62,63^ reported LBR at threshold value < 18.9 ng/ml, < 31.4 ng/ml, < 125.8 ng/ml, < 47.2 ng/, < 78.6 ng/ml, < 125.8 ng/ml, < 36.1 ng/ml and < 79.2 ng/ml. No studies reported on OPR, three studies^61–63^ reported CPR and MR. The threshold value used were < 18.9 ng/ml, < 31.4 ng/ml, < 125.8 ng/ml, < 25.2 ng/ml, < 47.2 ng/ml, < 78.6 ng/ml, < 125.8 ng/ml, < 36.1 ng/ml and < 79.2 ng/ml (Fig. 5). Data from various threshold values were summarised in Supplementary Table S4.

#### Outcomes: FET cycle

##### A. Before ovulation in a natural FET cycle

One study^66^ reported LBR at threshold level > 1.0 ng/ml; one study^65^ reported OPR at threshold level > 1.57 ng/ml; two studies^65,66^ reported CPR and MR at threshold levels > 1.0 ng/ml and > 1.57 ng/ml. (Supplementary Table S3).

##### B. Luteal phase

**Fig. 6:**
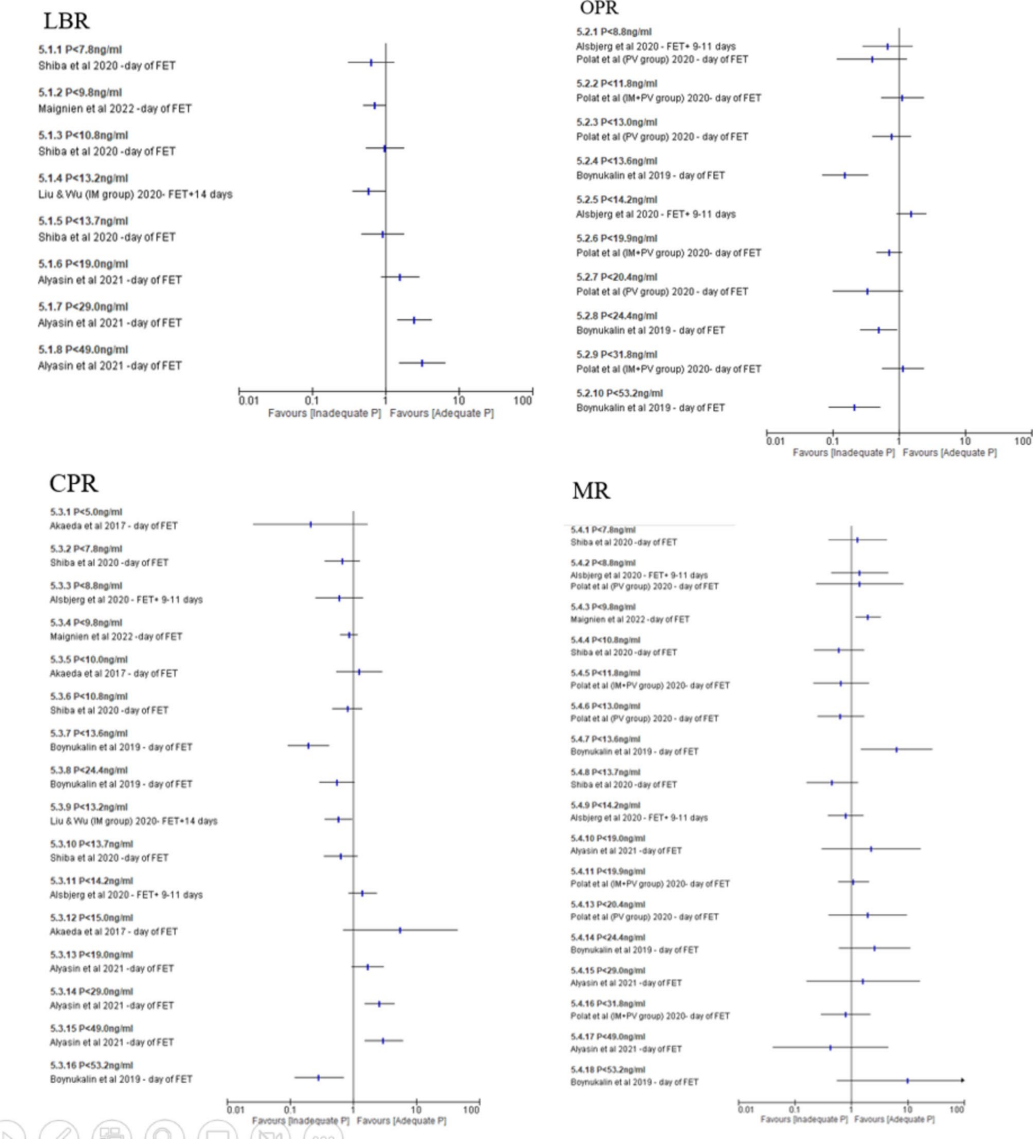
Inadequate vs adequate P during luteal phase of medicated FET cycle; outcome: LBR, OPR, CPR and MR. Forest plot of comparison on single studies between adequate progesterone group and inadequate progesterone group on LBR, OPR, CPR and MR during luteal phase in medicated FET cycle. *CPR* clinical pregnancy rate, *FET* frozen embryo transfer, *LBR* live birth rate, *MR* miscarriage rate, OPR ongoing pregnancy rate*, P* progesterone.

In medicated FET cycles, four studies^70,72–74^ reported on LBR, three studies^68,69,71^ reported on OPR, seven studies^67–70,72–74^ reported on CPR and six studies^68,69,71–74^ reported on MR at various threshold values (Fig. 6). There were no similarities between the threshold values used and wide variation of the timing of progesterone measurement. Data from various threshold values were summarised in Supplementary Table S5. In both natural cycle and medicated FET cycle, Melo et al.^75^ reported LBR, CPR and MR as summarised in Supplementary Table S6.

## Discussion

### Main findings

We set to examine whether serum progesterone level at different stages of the treatment impact on the outcomes. In controlled ovarian stimulation cycle with fresh embryo transfer, elevated progesterone at baseline did not impact on LBR/CPR. EP on the day of ovulation trigger in all studies (both D3 and D5) is associated with a decreased LBR/OPR/CPR and no significant difference in miscarriage. However, in a subgroup analysis, EP at ovulation trigger is associated with a lower LBR/CPR when D3 embryos were transferred. EP did not impact LBR/CPR when D5 embryos were transferred. There were insufficient studies to allow meaningful analysis for EP on the day of oocyte retrieval and on the day of embryo transfer.

In FET cycles, as the studies were heterogeneous with various threshold levels used and timing of serum progesterone monitoring, we were unable to combine the data in a meaningful way to give a definitive answer.

**Fig. 7:**
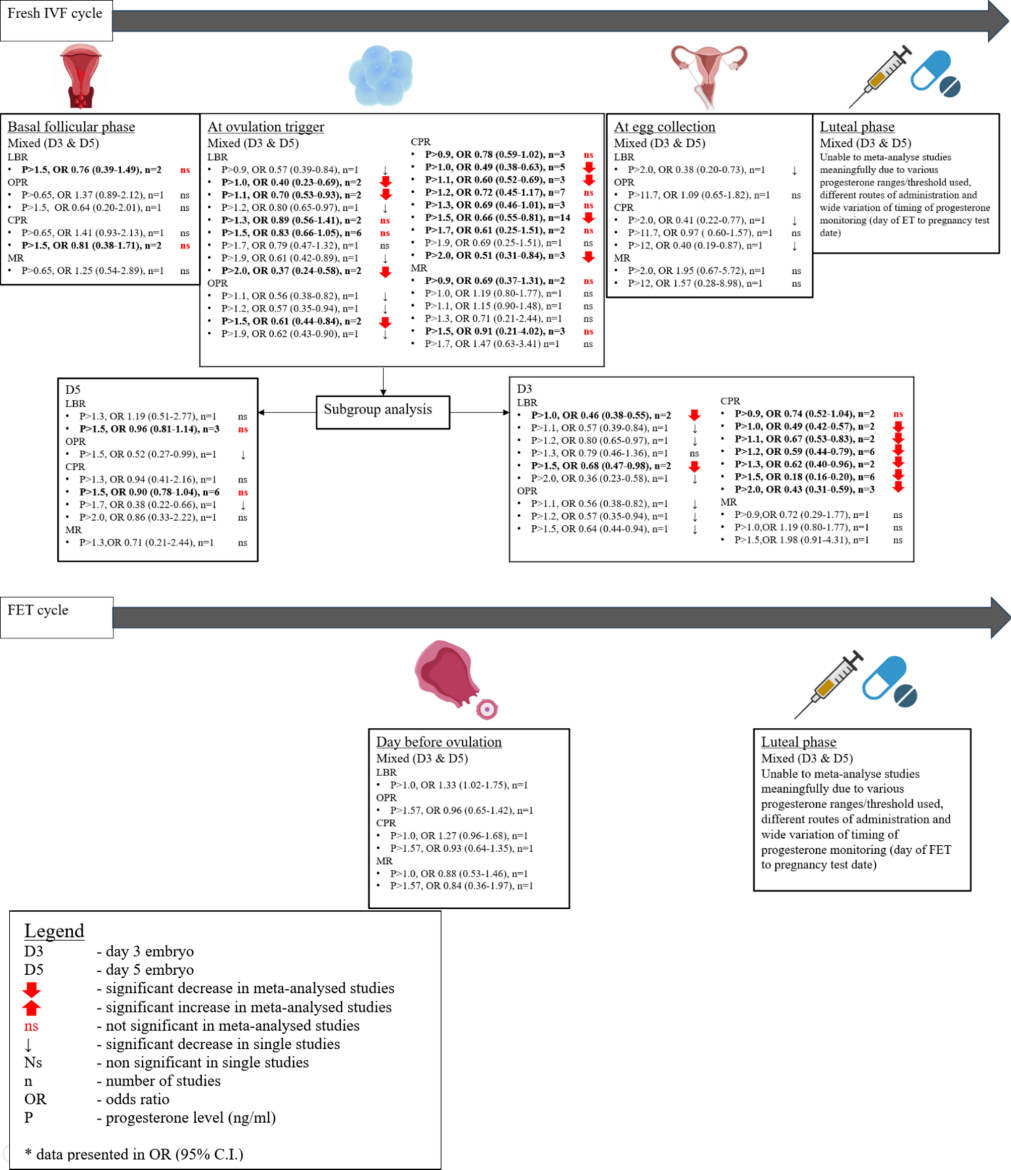
Summary of pregnancy outcomes. A summary of pregnancy outcomes according to different timing of progesterone monitoring at different threshold levels. *CPR* clinical pregnancy rate, *EP* elevated progesterone, *FET* frozen embryo transfer, *LBR* live birth rate, *MR* miscarriage rate, *NEP* non-elevated progesterone, *OPR* ongoing pregnancy rate.

We have provided a summary of our results in Fig. 7.

### Meaning of the findings

Whilst multiple theories exist to explain why EP in COS is harmful, the real mechanism is unknown. Recent data suggests that after the hCG trigger, progesterone levels peak from day 2 to 4 days after egg retrieval, at a level 10 times higher than natural cycles and several fold higher than levels achieved with luteal phase support, and the progesterone levels fall rapidly (in hours) after the peak^76^. One possible explanation for the harmful impact of EP may be related to the transient detrimental impact of acute progesterone withdrawal on the endometrium^77^, an event salvageable to an extent with progesterone replacement^78^; the latter theoretically having more impact on day 3 rather than day 5 embryos as the endometrium recovers. Another explanation of our findings may simply relate to the more robust nature of the blastocyst. Even after introducing the freeze-all strategy after a cycle of elevated progesterone, twenty-one studies evaluated the effect of elevated progesterone in ART cycles with nineteen studies using cleavage-stage embryos and twelve studies using blastocyst embryo transfer.

Hence findings must be viewed with caution.

### Strength of this review

This systematic review with meta-analysis examined the impact of serum progesterone measurement in all the phases of ART in fresh and frozen cycles. The strength of this systematic review is the inclusion of a large number of studies (64 studies, N = 57,988 women) and the fact that we only included studies analysing data per woman rather than per cycle, which reduces confounding. To the best of our knowledge, this is the first systematic review looking at progesterone elevation at different stages of cycle and a subgroup analysis based on day 3 and day 5 embryos.

### Limitations

The limitation of this review is that the included studies were observational studies and thus are subjected to confounding and prone to bias. Studies which are non-English and studies with multiple observations were also excluded. The included studies also exhibit increased clinical heterogeneity given the wide variation of thresholds adopted by studies with different types of responders, protocols, stages of embryo development transfer and in fresh and frozen cycles with variable outcomes; we are unable to perform meta-analysis in a meaningful way for several of our comparisons. Attempts were made to contact authors for their raw data, however we did not have any response.

We acknowledged the limitations on including studies post year 2000 and the exclusion of studies with multiple observations, which may result in inherent publication bias and some confounding factors uncontrolled for.

### Comparison with existing meta-analyses

One meta-analysis examined serum progesterone levels at baseline^3^, which reported a 15% reduction of OPR in women with EP. However, interventional studies were included, in which the initiation of COS was delayed until progesterone was normalised. In contrast, our current meta-analysis included studies that started the COS regardless of the progesterone level at baseline. We found similar LBR in both groups.

Three meta-analyses evaluated the association of EP on the day of HCG trigger^4–6^. Venetis et al.^5^ found no association between EP and CPR, whilst Kolibianakis et al.^4^ reported a significant decrease in CPR in the EP group. Venetis et al.^6^ later reported a lower pregnancy rate in women with EP on the day of the trigger during the fresh embryo transfer cycle but did not find any association in subsequent FET cycles. Subsequent studies published after that included pregnancy outcomes from blastocyst embryo transfer^32,34,38,39,44,55^ showing mixed results with some studies showing poorer pregnancy outcomes^32,34,55^ and some studies showing similar^38,44^ or better^39^ pregnancy outcomes. The very real change in practice with most clinics not transferring fresh embryos in the event of elevated progesterone means that the evidence regarding the effect of elevated progesterone in blastocysts transfers is quite limited and prone to publication bias.

One recent meta-analysis^7^ assessed PV progesterone supplementation in medicated FET cycles and reported a higher live birth rate in women with a higher progesterone level when compared to lower progesterone level (P < 10 ng/ml). While a minimum serum concentration of progesterone is required, the optimal level remains to be determined.

### Clinical implications

We do not recommend doing progesterone testing at baseline. While testing on the day of trigger is widely practiced, it needs to be interpreted with caution. It would be good practice for clinics to audit their clinical data to make decisions on the level of progesterone cut-off. In addition, progesterone levels should contribute but should not be the only decision making factor for freeze all. While most data on frozen embryo transfer comes from the medicated cycle, a shift to the natural cycle due to data on obstetric and perinatal outcomes may make progesterone testing a non question going forward.

### Implications for future research

Future research should take a two-step approach. First, the normal variation of serum progesterone levels in the normal population undergoing ART treatment in both fresh and frozen cycles should be determined. Second, by taking knowledge and experience gained from AMH testing, researchers can facilitate the creation of a nomogram on which future treatment and research can be based. Results from interventional trials can then advise if progesterone monitoring in a routine manner can be clinically beneficial.

## Conclusion

This review shows that there is no evidence that EP at baseline and oocyte retrieval impacts LBR. EP at the time of ovulation trigger decreases LBR only when day 3 embryo transfers are included; EP did not impact LBR with D5 embryos. Significant heterogeneity exists in the studies examined, and the evidence is of very low to low quality (Supplementary Table S7). Further good quality studies are needed to give a definitive answer.

## Electronic supplementary material

Below is the link to the electronic supplementary material.

Supplementary Material 1

## Data Availability

Data can be shared according to data protection legislation upon reasonable request to Y.C.L.
