# Supplementary material for: Progesterone level in assisted reproductive technology: a systematic review and meta-analysis
Source: Sci Rep. 2024 Dec 28;14:30826. doi: 10.1038/s41598-024-81539-z (PMC11681007; doi:10.1038/s41598-024-81539-z)
Supplement: Supplementary file 1 — Supplementary Material 1 [file 41598_2024_81539_MOESM1_ESM.docx]

**Supplementary Table S1: Keywords used in each database search**

| **No.** | **Database** | **Search terms** | **Results** |
| --- | --- | --- | --- |
| 1 | Pubmed | ("Progesterone"[Mesh]) OR "Progesterone/blood"[Mesh] AND "Reproductive Techniques, Assisted"[Mesh] OR "Fertilization in Vitro"[Mesh] OR "Sperm Injections, Intracytoplasmic"[Mesh] OR "Embryo Transfer"[Mesh] AND "Pregnancy Outcome"[Mesh] | 7,143 |
| 2 | EMBASE | Progesterone monitoring OR progesterone OR progesterone blood AND assisted reproductive techniques OR in vitro fertilization OR intracytoplasmic sperm injection OR embryo transfer AND pregnancy outcome | 742 |
| 3 | MEDLINE | Progesterone monitoring OR progesterone OR progesterone blood AND assisted reproductive techniques OR in vitro fertilization OR intracytoplasmic sperm injection OR embryo transfer AND pregnancy outcome | 192 |
| 4 | CINAHL | Progesterone monitoring OR progesterone OR progesterone blood AND assisted reproductive techniques OR in vitro fertilization OR intracytoplasmic sperm injection OR embryo transfer AND pregnancy outcome | 160 |

**Supplementary Table S2: Assessment of Quality of Studies Using Newcastle-Otawa scale**

| Reference | Case-cohort represent-ative | Selection of non-exposed control | Ascertai-nment of exposure | Outcome negative at start | Compara-bility by design/ analysis | Outcome assessme-nt | Duration of follow-up | Adequacy of follow up |
| --- | --- | --- | --- | --- | --- | --- | --- | --- |
| Hamdine et al, 2014 | 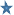 | 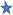 | 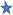 | 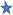 | 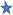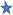 | 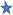 | 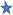 | 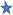 |
| Mahapatro & Radhakrishan, 2017 | 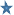 | 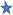 | 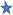 | 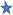 | 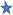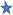 | 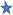 | 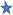 | 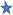 |
| Mutlu et al, 2017 | 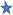 | 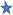 | 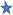 | 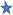 | 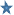 | 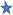 | 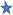 | 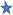 |
| Bosch et al, 2003 | 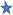 | 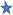 | 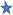 | 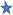 | 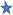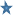 | 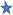 | 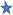 | 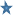 |
| Martinez et al, 2003 | 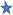 | 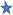 | 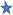 | 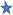 | 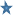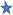 | 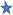 | 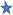 | 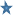 |
| Anderson et al, 2006 | 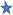 | 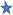 | 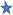 | 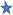 | 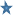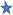 | 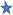 | 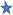 | 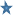 |
| Seow KM et al, 2007 | 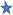 | 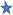 | 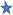 | 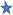 | 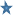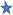 | 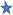 | 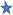 | 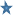 |
| Lee F et al, 2008 | 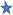 | 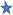 | 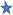 | 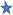 | 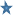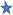 | 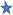 | 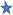 | 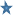 |
| Li R et al, 2008 | 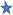 | 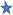 | 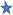 | 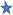 | 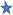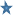 | 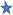 | 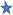 | 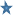 |
| Kiliçdag et al, 2009 | 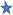 | 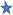 | 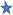 | 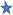 | 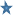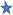 | 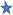 | 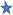 | 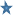 |
| Papanikolaou et al, 2009 | 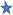 | 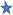 | 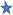 | 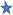 | 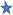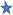 | 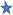 | 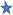 | 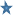 |
| Rezaee et al, 2009 | 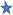 | 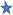 |  |  |  |  |  |  |
| Seow KM et al, 2010 |  |  |  |  |  |  |  |  |
| Elgindy, 2011 |  |  |  |  |  |  |  |  |
| Lahoud et al, 2011 |  |  |  |  |  |  |  |  |
| Yding Anderson et al, 2011 |  |  |  |  |  |  |  |  |
| Huang R et al, 2012 |  |  |  |  |  |  |  |  |
| Kyrou et al, 2012 |  |  |  |  |  |  |  |  |
| Papanikolaou et al, 2012 |  |  |  |  |  |  |  |  |
| Peng C et al, 2012 |  |  |  |  |  |  |  |  |
| Ochsenkuhn et al, 2012 |  |  |  |  |  |  |  |  |
| Wu Z et al, 2012 |  |  |  |  |  |  |  |  |
| Corti et al, 2013 |  |  |  |  |  |  |  |  |
| Griesinger et al, 2013 |  |  |  |  |  |  |  |  |
| Orvieto et al, 2013 |  |  |  |  |  |  |  |  |
| Papaleo et al, 2014 |  |  |  |  |  |  |  |  |
| Acet et al, 2015 |  |  |  |  |  |  |  |  |
| Huang P et al, 2015 |  |  |  |  |  |  |  |  |
| Huang Y et al, 2015 |  |  |  |  |  |  |  |  |
| Koo et al, 2015 |  |  |  |  |  |  |  |  |
| Singh et al, 2015 |  |  |  |  |  |  |  |  |
| Tsai Y et al, 2015 |  |  |  |  |  |  |  |  |
| Demir et al, 2016 |  |  |  |  |  |  |  |  |
| Healy et al, 2016 |  |  |  |  |  |  |  |  |
| Groenewoud et al, 2017 |  |  |  |  |  |  |  |  |
| Ashmita et al, 2018 |  |  |  |  |  |  |  |  |
| Simon et al, 2019 |  |  |  |  |  |  |  |  |
| Wu et al, 2019 |  |  |  |  |  |  |  |  |
| Lee C et al, 2020 |  |  |  |  |  |  |  |  |
| Yu Y et al, 2020 |  |  |  |  |  |  |  |  |
| Benmachiche et al, 2021 |  |  |  |  |  |  |  |  |
| Mahran et al, 2021 |  |  |  |  |  |  |  |  |
| Mirta et al, 2021 |  |  |  |  |  |  |  |  |
| Yang et al, 2021 |  |  |  |  |  |  |  |  |
| Jiang W et al, 2022 |  |  |  |  |  |  |  |  |
| Kong N et al, 2022 |  |  |  |  |  |  |  |  |
| Zhao et al, 2022 |  |  |  |  |  |  |  |  |
| Niu Z et al, 2008 |  |  |  |  |  |  |  |  |
| Nayak et al, 2014 |  |  |  |  |  |  |  |  |
| Tulic et al, 2020 |  |  |  |  |  |  |  |  |
| Kim et al, 2017 |  |  |  |  |  |  |  |  |
| Akaeda et al, 2019 |  |  |  |  |  |  |  |  |
| Thomsen et al, 2018 |  |  |  |  |  |  |  |  |
| Boynukalin et al, 2019 |  |  |  |  |  |  |  |  |
| Netter et al, 2019 |  |  |  |  |  |  |  |  |
| Alsbjerg et al, 2020 |  |  |  |  |  |  |  |  |
| Liu & Wu, 2020 |  |  |  |  |  |  |  |  |
| Polat et al, 2020 |  |  |  |  |  |  |  |  |
| Shiba et al, 2021 |  |  |  |  |  |  |  |  |
| Alyasin et al, 2021 |  |  |  |  |  |  |  |  |
| Maignien et al, 2022 |  |  |  |  |  |  |  |  |
| Melo et al, 2022 |  |  |  |  |  |  |  |  |
| Lee VC et al, 2014 |  |  |  |  |  |  |  |  |
| Wu D et al, 2022 |  |  |  |  |  |  |  |  |

**Supplementary Table S3**

| Study | P level  (ng/ml) | Elevated P | | Non-elevated P | | Outcome | OR (95% CI) |
| --- | --- | --- | --- | --- | --- | --- | --- |
|  |  | Events | Total | Events | Total |  |  |
| Basal follicular phase |  |  |  |  |  |  |  |
| Mutlu et al, 2017 | >0.65 | 45 | 139 | 84 | 325 | OPR | 1.37 (0.89-2.12) |
|  |  | 54 | 139 | 101 | 325 | CPR | 1.41 (0.93-2.13) |
|  |  | 9 | 139 | 17 | 325 | MR | 1.25 (0.54-2.89) |
| Hamdine et al, 2014 | >1.5 | 4 | 21 | 37 | 137 | OPR | 0.64 (0.20-2.01) |
| At ovulation trigger |  |  |  |  |  |  |  |
| Kilicdag et al, 2009 | >0.9 | 40 | 145 | 360 | 900 | LBR | 0.57 (0.39-0.84) |
|  |  | 40 | 145 | 366 | 900 | OPR | 0.56 (0.38-0.82) |
| Wu Z et al, 2012 | >1.0 | 47 | 318 | 74 | 583 | MR | 1.19 (0.80-1.77) |
| Yu Y et al, 2020 | >1.0 | 118 | 424 | 291 | 1161 | MR | 1.15 (0.90-1.48) |
| Huang R et al, 2012 | >1.2 | 172 | 627 | 624 | 1939 | LBR | 0.80 (0.65-0.97) |
| Anderson et al, 2006 | >1.2 | 22 | 126 | 155 | 573 | OPR | 0.57 (0.35-0.94) |
| Acet et al, 2015 | >1.3 | 4 | 33 | 11 | 68 | MR | 0.71 (0.21-2.44) |
| Lahoud et al, 2011 | >1.7 | 22 | 117 | 99 | 437 | LBR | 0.79 (0.47-1.32) |
|  |  | 10 | 34 | 29 | 131 | MR | 1.47 (0.63-3.41) |
| Tsai Y et al, 2015 | >1.9 | 40 | 180 | 422 | 1328 | LBR | 0.61 (0.42-0.89) |
|  |  | 42 | 180 | 436 | 1328 | OPR | 0.62 (0.43-0.90) |
|  |  | 57 | 180 | 533 | 1328 | CPR | 0.69 (0.50-0.96) |
| At egg collection |  |  |  |  |  |  |  |
| Tulic et al, 2020 | >2.0 | 30 | 91 | 41 | 73 | LBR | 0.38 (0.20-0.73) |
|  |  | 40 | 91 | 48 | 73 | CPR | 0.41 (0.22-0.77) |
|  |  | 10 | 40 | 7 | 48 | MR | 1.95 (0.67-5.72) |
| Niu Z et al, 2008 | >11.7 | 36 | 114 | 52 | 175 | OPR | 1.09 (0.65-1.82) |
|  |  | 44 | 114 | 69 | 175 | CPR | 0.97 (0.60-1.57) |
| Nayak et al, 2014 | >12.0 | 10 | 51 | 51 | 135 | CPR | 0.40 (0.19-0.87) |
|  |  | 2 | 10 | 7 | 51 | MR | 1.57 (0.28-8.98) |
| At ovulation trigger in modified NC-FET cycle | | | |  |  |  |  |
| Groenewoud et al, 2017 | >1.47 | 28 | 186 | 11 | 85 | LBR | 1.19 (0.56-2.52) |
|  |  | 28 | 186 | 13 | 85 | OPR | 0.98 (0.48-2.01) |
|  |  | 40 | 186 | 23 | 85 | CPR | 0.74 (0.41-1.34) |
| Before ovulation in natural FET cycle | | |  |  |  |  |  |
| Wu D et al, 2022 | >1.0 | 163 | 283 | 442 | 876 | LBR | 1.33 (1.02-1.75) |
|  |  | 187 | 283 | 531 | 876 | CPR | 1.27 (0.96-1.68) |
|  |  | 22 | 187 | 70 | 531 | MR | 0.88 (0.53-1.46) |
| Lee VC et al, 2014 | >1.57 | 51 | 161 | 146 | 449 | OPR | 0.96 (0.65-1.42) |
|  |  | 60 | 161 | 175 | 449 | CPR | 0.93 (0.64-1.35) |
|  |  | 8 | 60 | 27 | 175 | MR | 0.84 (0.36-1.97) |

Abbreviations: C.I., confidence interval; CPR, clinical pregnancy rate; MR, miscarriage rate; OPR, ongoing pregnancy rate; OR, odds ratio; P, progesterone

**Supplementary Table S4**

| Study | Route | Timing of P4 monitoring | P4 cutoff (ng/ml) | No of event/ total (n/N) | | Odds Ratio (95% CI) |
| --- | --- | --- | --- | --- | --- | --- |
|  |  |  |  | Inadequate P | Adequate P |  |
| LBR | | | | | | |
| Thomsen et al (early luteal phase), 2018 | PV | OPU +2/3  (day of ET) | <18.9 | 3/17 | 127/415 | 0.49 (0.14, 1.72) |
|  |  |  | <31.4 | 23/87 | 107/345 | 0.80 (0.47, 1.36) |
|  |  |  | <125.8 | 123/403 | 7/29 | 1.38 (0.57, 3.32) |
| Thomsen et al (mid-luteal phase), 2018 | PV | OPU + 5  (day of ET) | <47.2 | 16/34 | 57/136 | 1.23 (0.58, 2.62) |
|  |  |  | <78.6 | 41/78 | 32/92 | 2.08 (1.12, 3.85) |
|  |  |  | <125.8 | 58/117 | 15/53 | 2.49 (1.24, 0.86) |
| Netter et al, 2019 | oral | OPU +2/3  (day of ET) | <36.1 | 3/50 | 40/192 | 0.24 (0.07, 0.82) |
|  |  |  | <79.2 | 24/172 | 19/70 | 0.44 (0.22, 0.86) |
| CPR | | | | | | |
| Thomsen et al (early luteal phase), 2018 | PV | OPU +2/3  (day of ET) | <18.9 | 3/17 | 132/415 | 0.46 (0.13, 1.63) |
|  |  |  | <31.4 | 27/87 | 108/345 | 0.99 (0.59, 1.64) |
|  |  |  | <125.8 | 128/403 | 7/29 | 1.46 (0.61, 3.51) |
| Kim et al, 2017 | PV | OPU + 14 | <25.2 | 23/71 | 67/77 | 0.07 (0.03, 0.16) |
| Thomsen et al (mid-luteal phase), 2018 | PV | OPU + 5  (day of ET) | <47.2 | 16/34 | 60/136 | 1.13 (0.53, 2.39) |
|  |  |  | <78.6 | 43/78 | 33/92 | 2.20 (1.19, 4.07) |
|  |  |  | <125.8 | 60/117 | 16/53 | 2.43 (1.22, 4.85) |
| Netter et al, 2019 | oral | OPU +2/3  (day of ET) | <36.1 | 4/50 | 43/192 | 0.30 (0.10, 0.88) |
|  |  |  | <79.2 | 26/172 | 21/70 | 0.42 (0.21, 0.80) |
| MR | | | | | | |
| Thomsen et al (early luteal phase), 2018 | PV | OPU +2/3  (day of ET) | <18.9 | 2/5 | 46/178 | 1.91 (0.31, 11.81) |
|  |  |  | <31.4 | 13/40 | 35/143 | 1.49 (0.69, 3.19) |
|  |  |  | <125.8 | 43/171 | 5/12 | 0.47 (0.14, 1.56) |
| Kim et al, 2017 | PV | OPU + 14 | <25.2 | 48/71 | 10/77 | 13.98 (6.10, 32.06) |
| Thomsen et al (mid-luteal phase), 2018 | PV | OPU + 5  (day of ET) | <47.2 | 5/21 | 22/82 | 0.85 (0.28, 2.60) |
|  |  |  | <78.6 | 13/56 | 14/47 | 0.71 (0.30, 1.72) |
|  |  |  | <125.8 | 15/75 | 12/28 | 0.33 (0.13, 0.85) |
| Netter et al, 2019 | oral | OPU +2/3  (day of ET) | <36.1 | 5/9 | 17/60 | 3.16 (0.76, 13.21) |
|  |  |  | <79.2 | 16/42 | 6/27 | 2.15 (0.72, 6.47) |

Supplementary Table S5: Table of comparison between inadequate P group and adequate P group on LBR, CPR and MR during the luteal phase in fresh COS cycle

Abbreviations: COS, controlled ovarian stimulation; CPR, clinical pregnancy rate; EP, elevated progesterone; ET, embryo transfer; LBR, live birth rate; MR, miscarriage rate; NEP, non-elevated progesterone; OPU, oocyte pickup; P/P4, progesterone; PV, per vaginal

**Supplementary Table S5**

| Study | Route | Timing of P4 monitoring | P4 cutoff (ng/ml) | No of event/ total (n/N) | | Odds Ratio (95% CI) |
| --- | --- | --- | --- | --- | --- | --- |
|  |  |  |  | Inadequate P | Adequate P |  |
| LBR | | | | | | |
| Liu & Wu, 2020 | IM | FET + 14 | <13.15 | 43/131 | 59/131 | 0.60 (0.36, 0.98) |
| Shiba et al, 2021 | PV | Day of FET | <7.8 | 12/59 | 50/176 | 0.64 (0.32, 1.31) |
|  |  |  | <10.8 | 31/118 | 31/117 | 0.99 (0.55, 1.77) |
|  |  |  | <13.7 | 46/177 | 16/58 | 0.92 (0.47, 1.80) |
| Alyasin et al, 2021 | PV+IM | Day of FET | <19 | 27/64 | 61/194 | 1.59 (0.89, 2.85) |
|  |  |  | <29 | 57/129 | 31/129 | 2.50 (1.47, 4.26) |
|  |  |  | <49 | 77/194 | 11/64 | 3.17 (1.56, 6.45) |
| Maignien et al, 2022 | PV | Day of FET | <9.8 | 59/226 | 229/689 | 0.71 (0.51, 0.99) |
| OPR | | | | | | |
| Boynukalin et al, 2019 | IM | Day of FET | <13.6 | 11/42 | 88/126 | 0.15 (0.07, 0.34) |
|  |  |  | <24.4 | 43/85 | 56/83 | 0.49 (0.26, 0.92) |
|  |  |  | <53.2 | 65/127 | 34/41 | 0.22 (0.09, 0.52) |
| Alsbjerg et al, 2020 | PV+PR | FET + 9-11 | <8.8 | 9/25 | 97/214 | 0.68 (0.29, 1.60) |
|  |  |  | <14.2 | 60/121 | 46/118 | 1.54 (0.92, 2.57) |
| Polat et al (PV group), 2020 | PV | Day of FET | <8.75 | 4/14 | 65/129 | 0.39 (0.12, 1.32) |
|  |  |  | <12.95 | 32/71 | 37/72 | 0.78 (0.40, 1.50) |
|  |  |  | <20.42 | 59/129 | 10/14 | 0.34(0.10, 1.13) |
| Polat et al (PV + IM group), 2020 | PV+IM | Day of FET | <11.75 | 18/33 | 154/299 | 1.13 (0.55, 2.33) |
|  |  |  | <19.87 | 79/166 | 93/166 | 0.71 (0.46, 1.10) |
|  |  |  | <31.79 | 156/299 | 16/33 | 1.16 (0.56, 2.38) |
| CPR | | | | | | |
| Akaeda et al, 2017 | PV | Day of FET | <5 | 1/13 | 31/110 | 0.21 (0.03, 1.70) |
|  |  |  | <10 | 19/68 | 13/55 | 1.25 (0.55, 2.84) |
|  |  |  | <15 | 31/108 | 1/15 | 5.64 (0.71, 44.72) |
| Boynukalin et al, 2019 | IM | Day of FET | <13.6 | 15/42 | 93/126 | 0.20 (0.09, 0.42) |
|  |  |  | <24.4 | 49/85 | 59/83 | 0.55 (0.29, 1.05) |
|  |  |  | <53.2 | 74/127 | 34/41 | 0.29 (0.12, 0.70) |
| Alsbjerg et al, 2020 | PV+PR | FET + 9-11 days | <8.8 | 9/25 | 103/214 | 0.61 (0.26, 1.43) |
|  |  |  | <14.2 | 62/121 | 50/118 | 1.43 (0.86, 2.38) |
| Liu & Wu, 2020 | IM | FET + 14 | <13.15 | 55/131 | 72/131 | 0.59 (0.36, 0.97) |
| Shiba et al, 2021 | PV | Day of FET | <7.8 | 17/59 | 66/176 | 0.67 (0.36, 2.81) |
|  |  |  | <10.8 | 39/118 | 44/117 | 0.82 (0.48, 1.40) |
|  |  |  | <13.7 | 58/177 | 25/58 | 0.64 (0.35, 1.18) |
| Alyasin et al, 2021 | PV+IM | Day of FET | <19 | 29/64 | 63/194 | 1.72 (0.97, 3.07) |
|  |  |  | <29 | 60/129 | 32/129 | 2.64 (1.55, 4.47) |
|  |  |  | <49 | 80/194 | 12/64 | 3.04 (1.53, 6.06) |
| Maignien et al, 2022 | PV | Day of FET | <9.8 | 91/226 | 302/689 | 0.86 (0.64, 1.17) |
| MR | | | | | | |
| Boynukalin et al, 2019 | IM | Day of FET | <13.6 | 4/15 | 5/93 | 6.40 (1.49, 27.46) |
|  |  |  | <24.4 | 6/49 | 3/59 | 2.60 (0.62, 11.01) |
|  |  |  | <53.2 | 9/74 | 0/34 | 10.01 (0.57, 177.13) |
| Alsbjerg et al, 2020 | PV+PR | FET + 9-11 days | <8.8 | 5/14 | 38/135 | 1.42 (0.45, 4.50) |
|  |  |  | <14.2 | 22/82 | 21/67 | 0.80 (0.39, 1.63) |
| Polat et al (PV group), 2020 | PV | Day of FET | <8.75 | 2/6 | 23/88 | 1.41 (0.24, 8.24) |
|  |  |  | <12.95 | 9/41 | 16/53 | 0.65 (0.25, 1.67) |
|  |  |  | <20.42 | 23/82 | 2/12 | 1.95 (0.40, 9.58) |
| Polat et al (PV + IM group), 2020 | PV+IM | Day of FET | <11.75 | 4/23 | 50/208 | 0.67 (0.22, 2.05) |
|  |  |  | <19.87 | 27/111 | 27/120 | 1.11 (0.60, 2.04) |
|  |  |  | <31.79 | 48/209 | 6/22 | 0.80 (0.29, 2.14) |
| Shiba et al, 2021 | PV | Day of FET | <7.8 | 5/17 | 16/66 | 1.30 (0.40, 4.26) |
|  |  |  | <10.8 | 8/39 | 13/44 | 0.62 (0.22, 1.69) |
|  |  |  | <13.7 | 12/58 | 9/25 | 0.46 (0.16, 1.31) |
| Alyasin et al, 2021 | PV+IM | Day of FET | <19 | 2/29 | 2/63 | 2.26 (0.30, 16.89) |
|  |  |  | <29 | 3/60 | 1/32 | 1.63 (0.16, 16.36) |
|  |  |  | <49 | 3/80 | 1/12 | 0.43 (0.04, 4.49) |
| Maignien et al, 2022 | PV | Day of FET | <9.8 | 32/91 | 65/302 | 1.98 (1.19, 3.29) |

Supplementary Table S6: Table of comparison between inadequate P group and adequate P group on LBR, CPR and MR during the luteal phase in medicated HRT FET cycle

Abbreviations: CPR, clinical pregnancy rate; FET, frozen embryo transfer; IM, intramuscular; LBR, live birth rate; MR, miscarriage rate; P4, progesterone; PR, per rectal; PV, per vaginal

**Supplementary Table S6**

| Study | Route | Timing of P4 monitoring | P4 cutoff (ng/ml) | No of event/ total (n/N) | | Odds Ratio (95% CI) |
| --- | --- | --- | --- | --- | --- | --- |
|  |  |  |  | Inadequate P | Adequate P |  |
| LBR | | | | | | |
| Melo et al, 2022 | PV (NC FET)  PV or S/C or both (HRT FET) | Day of FET | <7.8 | 11/39 | 145/359 | 0.58 (0.28, 1.20) |
| CPR | | | | | | |
| Melo et al, 2022 |  | Day of FET | <7.8 | 12/39 | 162/359 | 0.54 (0.27, 1.10) |
|  |  |  |  |  |  |  |
| MR |  |  |  |  |  |  |
| Melo et al, 2022 |  | Day of FET | <7.8 | 8/19 | 57/202 | 1.85 (0.71, 4.84) |

Supplementary Table S7: Table of comparison between inadequate P group and adequate P group on LBR, CPR and MR during the luteal phase in natural and medicated FET cycle

Abbreviations: CPR, clinical pregnancy rate; FET, frozen embryo transfer; HRT, hormone replacement therapy; IM, intramuscular; LBR, live birth rate; MR, miscarriage rate; NC, natural cycle; P/P4, progesterone; PV, per vaginal; S/C, subcutaneous

**Summary points based on GRADE assessment (Supplementary Table S7)**

| **Monitoring elevated progesterone compared to non-elevated progesterone in basal progesterone** | | | | | | | | | | | |
| --- | --- | --- | --- | --- | --- | --- | --- | --- | --- | --- | --- |
| **Certainty assessment** | | | | | | | **Summary of findings** | | | | |
| **Participants (studies) Follow-up** | **Risk of bias** | **Inconsistency** | **Indirectness** | **Imprecision** | **Publication bias** | **Overall certainty of evidence** | **Study event rates (%)** | | **Relative effect (95% CI)** | **Anticipated absolute effects** | |
|  |  |  |  |  |  |  | **With non-elevated progesterone** | **With elevated progesterone** |  | **Risk with non-elevated progesterone** | **Risk difference with elevated progesterone** |
| **Live birth rate (assessed with: P>1.5ng/ml)** | | | | | | | | | | | |
| 309 (2 observational studies) | not serious | not serious | not serious | serious^a^ | none | ⨁◯◯◯ Very low | 74/253 (29.2%) | 14/56 (25.0%) | **OR 0.76** (0.39 to 1.49) | 292 per 1,000 | **53 fewer per 1,000** (from 154 fewer to 89 more) |
| **Clinical pregnancy rate (assessed with: P>1.5ng/ml)** | | | | | | | | | | | |
| 309 (2 observational studies) | serious^b^ | not serious | not serious | serious^a^ | none | ⨁◯◯◯ Very low | 106/253 (41.9%) | 23/56 (41.4%) | **OR 0.81** (0.38 to 1.71) | 419 per 1,000 | **50 fewer per 1,000** (from 204 fewer to 133 more) |

**CI:** confidence interval; **OR:** odds ratio

#### Explanations

a. number of events small and wide CI

b. Mixture of day 3 and day 5 embryos

| **Monitoring elevated progesterone compared to non-elevated progesterone in pre-trigger day in fresh cycle, mixed embryo (LBR)** | | | | | | | | | | | |
| --- | --- | --- | --- | --- | --- | --- | --- | --- | --- | --- | --- |
| **Certainty assessment** | | | | | | | **Summary of findings** | | | | |
| **Participants (studies) Follow-up** | **Risk of bias** | **Inconsistency** | **Indirectness** | **Imprecision** | **Publication bias** | **Overall certainty of evidence** | **Study event rates (%)** | | **Relative effect (95% CI)** | **Anticipated absolute effects** | |
|  |  |  |  |  |  |  | **With non-elevated progesterone** | **With elevated progesterone** |  | **Risk with non-elevated progesterone** | **Risk difference with elevated progesterone** |
| **Live birth rate - P>1.0ng/ml** | | | | | | | | | | | |
| 2805 (2 observational studies) | serious^a^ | not serious | serious^a^ | not serious | none | ⨁◯◯◯ Very low | 506/1585 (31.9%) | 227/1220 (18.6%) | **OR 0.40** (0.23 to 0.69) | 319 per 1,000 | **161 fewer per 1,000** (from 222 fewer to 75 fewer) |
| **Live birth rate - P>1.1ng/ml** | | | | | | | | | | | |
| 3186 (2 observational studies) | serious^b^ | not serious | not serious | not serious | none | ⨁◯◯◯ Very low | 899/2147 (41.9%) | 329/1039 (31.7%) | **OR 0.70** (0.53 to 0.93) | 419 per 1,000 | **84 fewer per 1,000** (from 142 fewer to 18 fewer) |
| **Live birth rate - P>1.3ng/ml** | | | | | | | | | | | |
| 429 (2 observational studies) | serious^c^ | not serious | serious^d^ | serious^e^ | none | ⨁◯◯◯ Very low | 112/315 (35.6%) | 38/114 (33.3%) | **OR 0.89** (0.56 to 1.41) | 356 per 1,000 | **26 fewer per 1,000** (from 120 fewer to 82 more) |
| **Live birth rate - P>1.5ng/ml** | | | | | | | | | | | |
| 8170 (6 observational studies) | serious^b,c^ | serious^f^ | not serious | not serious | none | ⨁◯◯◯ Very low | 2483/6838 (36.3%) | 362/1332 (27.2%) | **OR 0.83** (0.66 to 1.05) | 363 per 1,000 | **42 fewer per 1,000** (from 90 fewer to 11 more) |
| **Live birth rate - P>2.0ng/ml** | | | | | | | | | | | |
| 2257 (2 observational studies) | serious^b^ | not serious | not serious | not serious | none | ⨁◯◯◯ Very low | 492/2051 (24.0%) | 23/206 (11.2%) | **OR 0.37** (0.24 to 0.58) | 240 per 1,000 | **135 fewer per 1,000** (from 169 fewer to 85 fewer) |

**CI:** confidence interval; **OR:** odds ratio

#### Explanations

a. Low responder in 1 study

b. Mixed stage embryos used in same studies

c. Mixed stage embryos used in different studies

d. High responder in 1 study

e. small sample size, wide CI

f. Large variation in effect, no overlap of CI, I^2^ high, p<0.05

| **Monitoring elevated progesterone compared to non-elevated progesterone in pre-trigger day in fresh cycle, mixed embryo (OPR)** | | | | | | | | | | | |
| --- | --- | --- | --- | --- | --- | --- | --- | --- | --- | --- | --- |
| **Certainty assessment** | | | | | | | **Summary of findings** | | | | |
| **Participants (studies) Follow-up** | **Risk of bias** | **Inconsistency** | **Indirectness** | **Imprecision** | **Publication bias** | **Overall certainty of evidence** | **Study event rates (%)** | | **Relative effect (95% CI)** | **Anticipated absolute effects** | |
|  |  |  |  |  |  |  | **With non-elevated progesterone** | **With elevated progesterone** |  | **Risk with non-elevated progesterone** | **Risk difference with elevated progesterone** |
| **Ongoing pregnancy rate - P>1.5ng/ml** | | | | | | | | | | | |
| 2070 (2 observational studies) | serious^a^ | not serious | not serious | not serious | none | ⨁◯◯◯ Very low | 633/1853 (34.2%) | 56/217 (25.8%) | **OR 0.61** (0.44 to 0.84) | 342 per 1,000 | **101 fewer per 1,000** (from 156 fewer to 38 fewer) |

**CI:** confidence interval; **OR:** odds ratio

#### Explanations

a. Mixed embryo stage in different studies

| **Monitoring elevated progesterone compared to non-elevated progesterone in pre-trigger day in fresh cycle, mixed embryo (CPR)** | | | | | | | | | | | |
| --- | --- | --- | --- | --- | --- | --- | --- | --- | --- | --- | --- |
| **Certainty assessment** | | | | | | | **Summary of findings** | | | | |
| **Participants (studies) Follow-up** | **Risk of bias** | **Inconsistency** | **Indirectness** | **Imprecision** | **Publication bias** | **Overall certainty of evidence** | **Study event rates (%)** | | **Relative effect (95% CI)** | **Anticipated absolute effects** | |
|  |  |  |  |  |  |  | **With non-elevated progesterone** | **With elevated progesterone** |  | **Risk with non-elevated progesterone** | **Risk difference with elevated progesterone** |
| **Clinical pregnancy rate - P>0.9ng/ml** | | | | | | | | | | | |
| 1238 (3 observational studies) | serious^a^ | not serious | serious | not serious | none | ⨁◯◯◯ Very low | 322/725 (44.4%) | 195/513 (38.0%) | **OR 0.78** (0.59 to 1.02) | 444 per 1,000 | **60 fewer per 1,000** (from 124 fewer to 5 more) |
| **Clinical pregnancy rate - P>1.0ng/ml** | | | | | | | | | | | |
| 4204 (5 observational studies) | serious^a,b^ | serious^c^ | serious^b^ | not serious | none | ⨁◯◯◯ Very low | 1025/2312 (44.3%) | 532/1892 (28.1%) | **OR 0.49** (0.38 to 0.63) | 443 per 1,000 | **163 fewer per 1,000** (from 211 fewer to 109 fewer) |
| **Clinical pregnancy rate - P>1.1ng/ml** | | | | | | | | | | | |
| 4969 (3 observational studies) | not serious | not serious | not serious | not serious | none | ⨁⨁◯◯ Low | 1732/3678 (47.1%) | 474/1291 (36.7%) | **OR 0.60** (0.52 to 0.69) | 471 per 1,000 | **123 fewer per 1,000** (from 155 fewer to 90 fewer) |
| **Clinical pregnancy rate - P>1.2ng/ml** | | | | | | | | | | | |
| 1319 (7 observational studies) | not serious | serious^c^ | not serious | serious^d^ | none | ⨁◯◯◯ Very low | 351/811 (43.3%) | 185/508 (36.4%) | **OR 0.72** (0.45 to 1.17) | 433 per 1,000 | **78 fewer per 1,000** (from 177 fewer to 39 more) |
| **Clinical pregnancy rate - P>1.3ng/ml** | | | | | | | | | | | |
| 732 (3 observational studies) | serious^e^ | not serious | not serious | not serious | none | ⨁◯◯◯ Very low | 186/553 (33.6%) | 49/179 (27.4%) | **OR 0.69** (0.46 to 1.01) | 336 per 1,000 | **77 fewer per 1,000** (from 147 fewer to 2 more) |
| **Clinical pregnancy rate - P>1.5ng/ml** | | | | | | | | | | | |
| 22901 (14 observational studies) | serious^a,e^ | serious^c^ | not serious | not serious | none | ⨁◯◯◯ Very low | 9729/20243 (48.1%) | 960/2658 (36.1%) | **OR 0.66** (0.55 to 0.81) | 481 per 1,000 | **101 fewer per 1,000** (from 143 fewer to 52 fewer) |
| **Clinical pregnancy rate - P>1.7ng/ml** | | | | | | | | | | | |
| 1700 (2 observational studies) | serious^a^ | serious^c^ | not serious | serious^d^ | none | ⨁◯◯◯ Very low | 572/1503 (38.1%) | 51/197 (25.9%) | **OR 0.61** (0.25 to 1.51) | 381 per 1,000 | **108 fewer per 1,000** (from 247 fewer to 101 more) |
| **Clinical pregnancy rate - P>2.0ng/ml** | | | | | | | | | | | |
| 2073 (3 observational studies) | not serious | serious^c^ | not serious | not serious | none | ⨁◯◯◯ Very low | 658/1783 (36.9%) | 75/290 (25.9%) | **OR 0.51** (0.31 to 0.84) | 369 per 1,000 | **139 fewer per 1,000** (from 216 fewer to 40 fewer) |

**CI:** confidence interval; **OR:** odds ratio

#### Explanations

a. Mixed stage embryos used in same study

b. specific population excluded

c. No overlap in CI, I^2^ high

d. wide CI

e. Mixed stage embryos used in different studies

| **Monitoring elevated progesterone compared to non-elevated progesterone in pre-trigger day in fresh cycle, mixed embryo (MR)** | | | | | | | | | | | |
| --- | --- | --- | --- | --- | --- | --- | --- | --- | --- | --- | --- |
| **Certainty assessment** | | | | | | | **Summary of findings** | | | | |
| **Participants (studies) Follow-up** | **Risk of bias** | **Inconsistency** | **Indirectness** | **Imprecision** | **Publication bias** | **Overall certainty of evidence** | **Study event rates (%)** | | **Relative effect (95% CI)** | **Anticipated absolute effects** | |
|  |  |  |  |  |  |  | **With non-elevated progesterone** | **With elevated progesterone** |  | **Risk with non-elevated progesterone** | **Risk difference with elevated progesterone** |
| **Miscarriage rate - P>0.9ng/ml** | | | | | | | | | | | |
| 800 (2 observational studies) | serious^a^ | not serious | serious^b^ | serious^c^ | none | ⨁◯◯◯ Very low | 30/480 (6.3%) | 17/320 (5.3%) | **OR 0.69** (0.37 to 1.31) | 63 per 1,000 | **19 fewer per 1,000** (from 38 fewer to 18 more) |
| **Miscarriage rate - P>1.5ng/ml** | | | | | | | | | | | |
| 1780 (3 observational studies) | serious^a^ | very serious^d^ | not serious | serious^c^ | none | ⨁◯◯◯ Very low | 136/1621 (8.4%) | 21/159 (13.2%) | **OR 0.91** (0.21 to 4.02) | 84 per 1,000 | **7 fewer per 1,000** (from 65 to 185 more) |

**CI:** confidence interval; **OR:** odds ratio

#### Explanations

a. Mixed stage embryos used in a study

b. different population

c. wide CI

d. No overlap in CI, I^2^ high, p<0.05

| **Monitoring elevated progesterone compared to non-elevated progesterone in pre-trigger day for blastocyst embryo in fresh cycle** | | | | | | | | | | | |
| --- | --- | --- | --- | --- | --- | --- | --- | --- | --- | --- | --- |
| **Certainty assessment** | | | | | | | **Summary of findings** | | | | |
| **Participants (studies) Follow-up** | **Risk of bias** | **Inconsistency** | **Indirectness** | **Imprecision** | **Publication bias** | **Overall certainty of evidence** | **Study event rates (%)** | | **Relative effect (95% CI)** | **Anticipated absolute effects** | |
|  |  |  |  |  |  |  | **With non-elevated progesterone** | **With elevated progesterone** |  | **Risk with non-elevated progesterone** | **Risk difference with elevated progesterone** |
| **Day 5 with LBR - P>1.5ng/ml** | | | | | | | | | | | |
| 5174 (3 observational studies) | not serious | serious^a^ | not serious | serious^b^ | none | ⨁◯◯◯ Very low | 1035/4174 (24.8%) | 241/1000 (24.1%) | **OR 0.96** (0.81 to 1.14) | **Low** | |
|  |  |  |  |  |  |  |  |  |  | 248 per 1,000 | **8 fewer per 1,000** (from 37 fewer to 25 more) |
| **Day 5 with CPR - P>1.5ng/ml** | | | | | | | | | | | |
| 5705 (6 observational studies) | not serious | serious^a^ | not serious | not serious | none | ⨁◯◯◯ Very low | 2329/4572 (50.9%) | 441/1133 (38.9%) | **OR 0.90** (0.78 to 1.04) | 509 per 1,000 | **26 fewer per 1,000** (from 62 fewer to 10 more) |

**CI:** confidence interval; **OR:** odds ratio

#### Explanations

a. No overlap in CI, I^2^ high, P>0.05

b. wide CI
